# Defective podocyte insulin signalling through p85-XBP1 promotes ATF6-dependent maladaptive ER-stress response in diabetic nephropathy

**DOI:** 10.1038/ncomms7496

**Published:** 2015-03-10

**Authors:** Thati Madhusudhan, Hongjie Wang, Wei Dong, Sanchita Ghosh, Fabian Bock, Veera Raghavan Thangapandi, Satish Ranjan, Juliane Wolter, Shrey Kohli, Khurrum Shahzad, Florian Heidel, Martin Krueger, Vedat Schwenger, Marcus J. Moeller, Thomas Kalinski, Jochen Reiser, Triantafyllos Chavakis, Berend Isermann

**Affiliations:** 1 Institute of Clinical Chemistry and Pathobiochemistry, Medical Faculty, Otto-von-Guericke University Magdeburg, Magdeburg 39120, Germany; 2 Department of Cardiology, Tongji Hospital, Tongji Medical College, Huazhong University of Science and Technology, 430030 Wuhan, China; 3 Department of Internal Medicine, Hematology and Oncology, Medical Faculty, Otto-von-Guericke University Magdeburg, Magdeburg 39120, Germany; 4 University of Health Sciences, Khayaban-e-Jamia Punjab, Lahore 54600, Pakistan; 5 Institute of Anatomy, University Leipzig, Liebigstrasse 13, 04103 Leipzig, Germany; 6 Internal Medicine I and Division of Nephrology, University of Heidelberg, Heidelberg 69120, Germany; 7 Division of Nephrology and Immunology, University Hospital of the RWTH Aachen University of Technology, Aachen 52074, Germany; 8 Institute of Pathology, Medical Faculty, Otto-von-Guericke University Magdeburg, Magdeburg 39120, Germany; 9 Division of Medicine, Rush University, Chicago, Illinois 60612, USA; 10 Department of Clinical Pathobiochemistry and Institute for Clinical Chemistry and Laboratory Medicine, Technische Universität Dresden, Dresden 01307, Germany

## Abstract

Endoplasmic reticulum (ER) stress is associated with diabetic nephropathy (DN), but its pathophysiological relevance and the mechanisms that compromise adaptive ER signalling in podocytes remain unknown. Here we show that nuclear translocation of the transcription factor spliced X-box binding protein-1 (sXBP1) is selectively impaired in DN, inducing activating transcription factor-6 (ATF6) and C/EBP homology protein (CHOP). Podocyte-specific genetic ablation of XBP1 or inducible expression of ATF6 in mice aggravates DN. sXBP1 lies downstream of insulin signalling and attenuating podocyte insulin signalling by genetic ablation of the insulin receptor or the regulatory subunits phosphatidylinositol 3-kinase (PI3K) p85α or p85β impairs sXBP1 nuclear translocation and exacerbates DN. Corroborating our findings from murine DN, the interaction of sXBP1 with p85α and p85β is markedly impaired in the glomerular compartment of human DN. Thus, signalling via the insulin receptor, p85, and XBP1 maintains podocyte homeostasis, while disruption of this pathway impairs podocyte function in DN.

## Abstract (web summary)

Diabetic kidney disease is associated with ER stress in podocytes. Here the authors use various genetically modified mouse models to study ER-stress-related signalling pathways and propose a mechanistic framework that links insulin signalling with ER stress in podocytes of diabetic mice.

## Graphical abstract

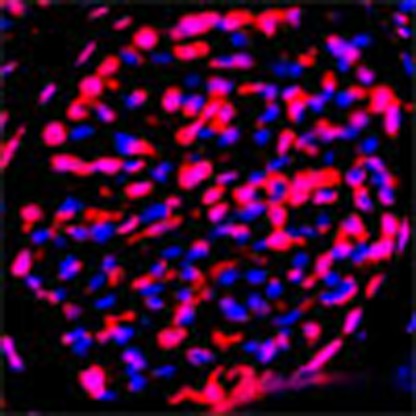

Diabetic nephropathy (DN) is the major cause of end-stage renal disease, and its incidence is continuously rising worldwide^1^,^2^. However, the underlying pathophysiological mechanisms remain incompletely understood, hampering the development of new therapeutic approaches. Recently, dysfunction of the endoplasmic reticulum (ER) has been linked with DN. Chemical chaperons such as tauroursodeoxycholic acid (TUDCA) or phenylbutyric acid, which enhance the ER-adaptive response, alleviate experimental DN^3^,^4^,^5^, implying that a maladaptive ER response is mechanistically linked with DN and that targeting the ER may be a promising therapeutic approach in DN. However, the mechanisms causing ER dysfunction in DN remain unknown precluding the design of specific therapeutic approaches.

Within the ER, a high demand for proper protein synthesis and folding can result in the accumulation of unfolded or misfolded proteins, resulting in ER stress and triggering the unfolded protein response (UPR). The UPR is a complex, yet highly coordinated programme, aiming to restore ER homeostasis either through proper folding of misfolded proteins via chaperons or degradation of these proteins^6^. While this adaptive process is frequently beneficial in acute diseases, prolonged or persistent activation of the UPR may be detrimental in chronic diseases. Thus, a relevance of ER stress has been demonstrated not only in DN, but also in obesity, insulin resistance, type 2 diabetes mellitus and atherosclerosis^7^,^8^,^9^.

Typically toxin-mediated impairment of ER protein folding activates all three major pathways initiated by ER transmembrane proteins IRE1 (Inositol-requiring enzyme 1), PERK (double-stranded RNA-activated protein kinase (PKR)-like ER kinase) and ATF6 collectively termed as UPR^6^,^10^. However, in a disease state these pathways are differentially or selectively regulated and each of these pathways have been implicated as either protective or pathological in diabetes mellitus or its complications^8^. The most conserved UPR pathway, the IRE1/XBP1 branch, has been linked both to constitutive and inducible UPR^11^,^12^,^13^,^14^. The constitutive, IRE1/XBP1-dependent UPR is required to maintain cellular homeostasis and function, as illustrated by, for example, the impaired lipid and insulin metabolism in mice with targeted XBP1 inactivation^15^,^16^ or the spontaneous colitis in mice with intestinal epithelial cell-specific XBP1 inactivation^14^. Recent studies shed light on a selective transactivation mechanism of the IRE1/XBP1 pathway, operating independently of canonical IRE1α activation, in particular, interaction of p85 with sXBP1 promotes its nuclear translocation and activation^17^,^18^,^19^.

While ER stress has been clearly linked with DN, the specific regulation of the tripartite UPR in DN during the progression of diabetes, the pathophysiological consequences of insulin resistance and/or hyperglycaemia for the regulation of the UPR, and the specific molecular targets that provoke these selective changes are not known. Understanding the mechanisms through which the three branches of the UPR are specifically regulated in DN may provide insights into the mechanisms of maladaptive versus adaptive UPR responses in DN and thus lay ground for novel therapeutic targets^20^.

Within the current study, using a combination of *in vitro* and cell-specific *in vivo* models as well as analysis of human renal biopsies and gene expression database, we show that loss of sXBP1 and gain of ATF6 function is characteristic for and mechanistically linked to a maladaptive ER-stress response in humans and mice, respectively. More importantly, the current study identifies that insulin signalling in podocytes regulates homeostatic UPR, and disturbance of insulin signalling is causatively linked to the maladaptive ER-stress response in DN.

## Results

### Disparate regulation of tripartite UPR in mouse and human DN

**Figure 1:**
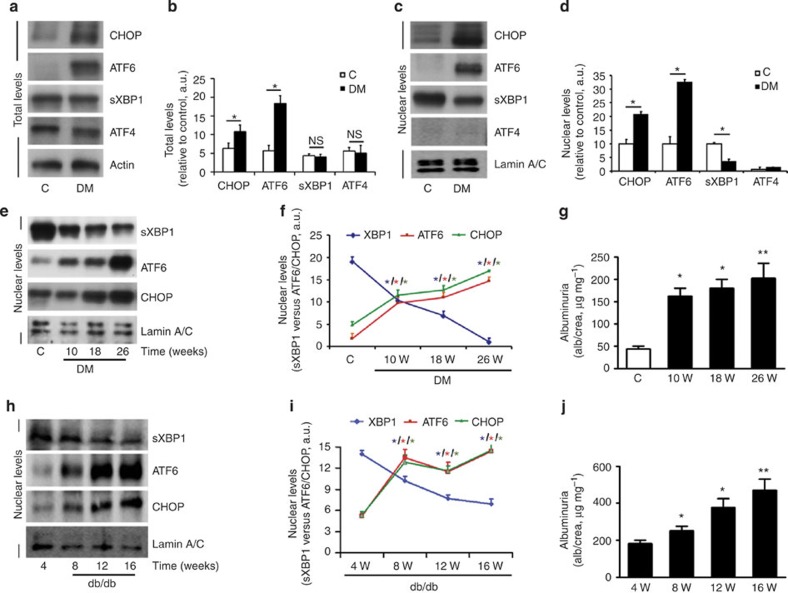
Hyperglycaemia differentially regulates the tripartite UPR in DN. (**a**–**d**) Representative immunoblots (**a**,**c**) and bar graphs (**b**,**d**) showing total (**a**,**b**) and nuclear (**c**,**d**) levels of ER transcription factors in renal cortex samples of wild-type control (C) and STZ-induced diabetic mice (DM). The 50 kDa form of ATF6 is shown in **a**. As loading controls, actin was used for total cell extracts (**a**) and lamin A/C for nuclear extracts (**c**). (*n=*6 mice per group). (**e**–**g**) Representative immunoblots (**e**) and line graph (**f**) showing nuclear levels of ER transcription factors in renal cortex samples (**e**,**f**) and albuminuria (**g**) in wild-type control (C) and STZ-induced diabetic (DM) mice at indicated time points post STZ administration; (*n=*8 mice per time point). (**h**–**j**) Representative immunoblots (**h**) and line graph (**i**) showing nuclear levels of ER transcription factors in renal cortex samples (**h**,**i**) and albuminuria (**j**) in db/db mice at ages indicated. (*n=*6 mice per time point). C=control mice without diabetes, open bars; DM=diabetes, black bars; Mean±s.e.m., *P<0.05, **P<0.01 (*t*-test, **b**,**d**, or ANOVA, **g**,**j**).

To delineate the mechanism controlling the UPR in DN, we employed two independent mouse models of DN. We used a mouse model of persistent hyperglycaemia induced (1) by streptozotocin (STZ model), reflecting insulinopenic type 1 DM, or (2) of insulin resistance and hyperglycaemia (db/db mice)^21^,^22^. Analyses of renal cortex samples in STZ-treated mice showed increased protein levels of transcription factor CHOP, indicating ER stress. Similarly, total levels of the active form of ATF6 (50 kDa) increased, while levels of sXBP1 and activating transcription factor-4 (ATF4) remained unchanged, establishing a distinct regulation of the three ER pathways in DN (Fig. 1a,b). Since a specific defect of hepatic sXBP1 nuclear translocation independently of total sXBP1 levels has been causally linked to ER stress, obesity and insulin resistance^17^,^18^, we next analysed cytosolic and nuclear extracts. This revealed a specific reduction of nuclear sXBP1, reflecting defective sXBP1 nuclear translocation in DN (Fig. 1c,d). While the nuclear levels of ATF6 (active form; 50 kDa) and CHOP increased, nuclear ATF4 was not induced excluding its involvement in this disease model (Fig. 1c,d and Supplementary Fig. 1).

To determine the role of ER stress in progression of DN, we systematically analysed the time-dependent activation of the three UPR branches at different stages of DN in both STZ-treated and db/db mouse models. A reduction in nuclear sXBP1 associated with increased nuclear ATF6 (50 kDa) and CHOP was already apparent at the earliest disease stages analysed (STZ-model: 10 weeks post STZ injection; db/db mice: age 8 weeks) and coincided with the onset of albuminuria (Fig. 1e–j and Supplementary Fig. 1). Given the lack of nuclear ATF4 induction we next determined the phosphorylation status of its upstream regulators PERK and eIF2α. Consistent with the low nuclear ATF4 levels, phosphorylation of PERK and eIF2α was not induced at different stages of DN (Supplementary Fig. 2a,b). Furthermore, no change in ATF4 mRNA levels was observed in renal cortex samples of DN when compared with wild-type non-diabetic controls (Supplementary Fig. 2c). Together, these data demonstrate that impaired nuclear translocation of sXBP1 is associated with increased levels of ATF6 and CHOP, while no involvement of the ATF4-related pathway was observed in DN.

**Figure 2:**
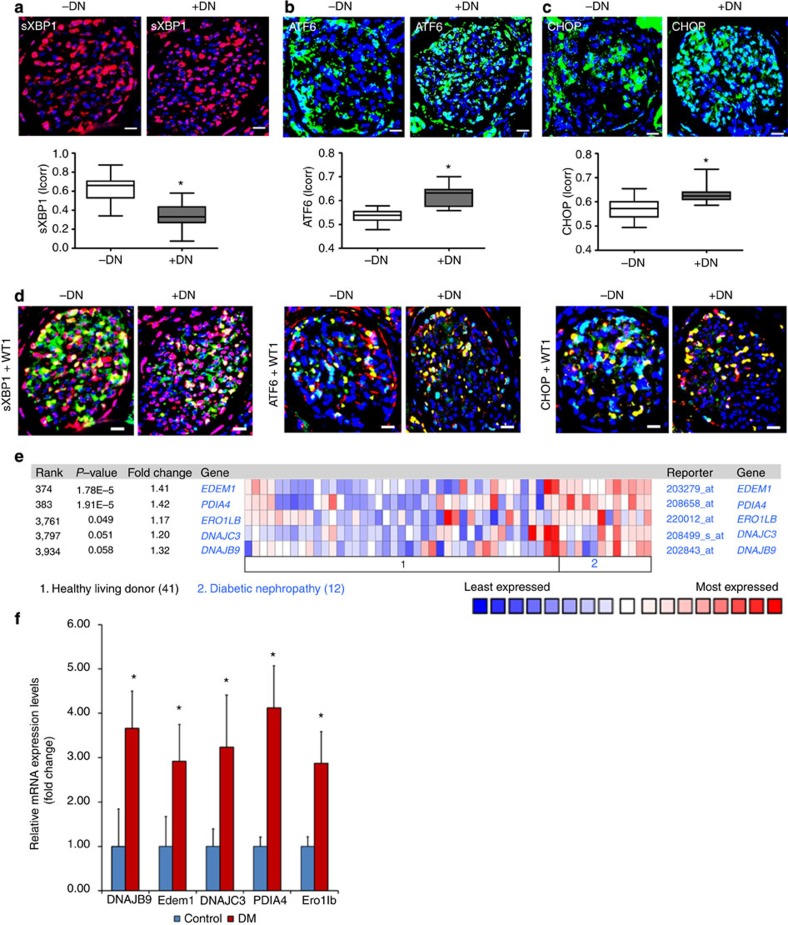
Maladaptive ER response in human and murine DN. (**a**–**c**) Representative confocal images showing sXBP1 (using an antibody detecting the spliced form of human XBP1, **a**), ATF6 (**b**) and CHOP (**c**) in human renal biopsies from diabetic patients without (–DN) or with (+DN) diabetic nephropathy and box plots showing Icorr-scores for nuclear localization. In human diabetic nephropathy, nuclear localization of sXBP1 was reduced primarily in renal glomeruli, while nuclear localization of ATF6 and CHOP was increased when compared with biopsies from healthy subjects. (*n=*5 human renal biopsies per group were analysed). Scale bar represents (**a**–**c**: 20 μm); *P<0.05, Wilcoxon test, **a**–**c**). (**d**) Representative immunofluorescent images of human renal biopsies obtained from diabetic patients without (–DN) or with (+DN) diabetic nephropathy, stained for sXBP1, ATF6, CHOP (red) and podocyte marker WT1 (green) with a nuclear counterstain (DAPI, blue). Single colour images of the merged images shown here are available in Supplementary Fig. 3b. (**e**) Analyses of UPR target genes indicating ER stress in DN: Heat map obtained from ‘Ju Podocyte’ group from Nephromine, showing glomerular-specific gene expression levels of UPR target genes in human healthy living donors (*n=*41) and patients with diabetic nephropathy (*n=*12). (**f**) Bar graph showing relative mRNA expression levels of UPR target genes in isolated mouse glomeruli from wild-type non-diabetic control mice (blue bars) and diabetic mice (26 weeks post-STZ treatment, red bars). The mRNA levels of the genes tested were normalized to 18S as an internal control; (*n=*5 mice per group were analysed). Mean±s.e.m. *P<0.05 (*t*-test, **f**).

To determine whether a disparate UPR occurs likewise in humans, we performed immunofluorescence analyses of renal biopsies of human subjects. Consistent with the above findings from murine DN, nuclear localization of sXBP1 (analysed by an antibody that specifically detects the spliced form of human XBP1) was reduced in renal biopsies of patients with DN primarily within the renal glomeruli, while nuclear localization of ATF6 and of CHOP was increased as compared with biopsies from healthy subjects (Fig. 2a–d and Supplementary Fig. 3). To further assess the pathophysiological relevance of ER-stress induction in human DN, we next analysed gene expression (mRNA levels) in a large human renal database (Nephromine)^23^,^24^. Within Nephromine database we analysed the gene expression (over expression) of UPR target genes in ‘Ju Podocyte’ database, which is a collection of gene expression profiling of 217 micro-dissected glomerular samples and 219 tubulointerstitial samples from chronic kidney disease (CKD) patients and healthy living donors. Gene expression analysis showed increased mRNA levels of UPR target genes known to be crucial for expanding the folding capacity of the ER (molecular chaperones: DNAJB9, DNAJC3, PDIA4, Ero1b) and for ER-associated degradation (ERAD: Edem1)^25^ in patients with DN when compared with healthy living donors (Fig. 2e). Consistent with these data, glomerular-specific mRNA levels of UPR-target genes were likewise upregulated in murine DN indicating ER stress (Fig. 2f). Collectively, these data establish a disparate regulation of the tripartite-UPR in human and murine DN. In addition, the relative abundance of nuclear sXBP1 in non-diabetic glomeruli indicates a constitutive-homeostatic function of this UPR signalling arm raising the question about the underlying regulatory mechanisms.

### Hyperglycaemia-induced ER stress is causally linked to DN

**Figure 3:**
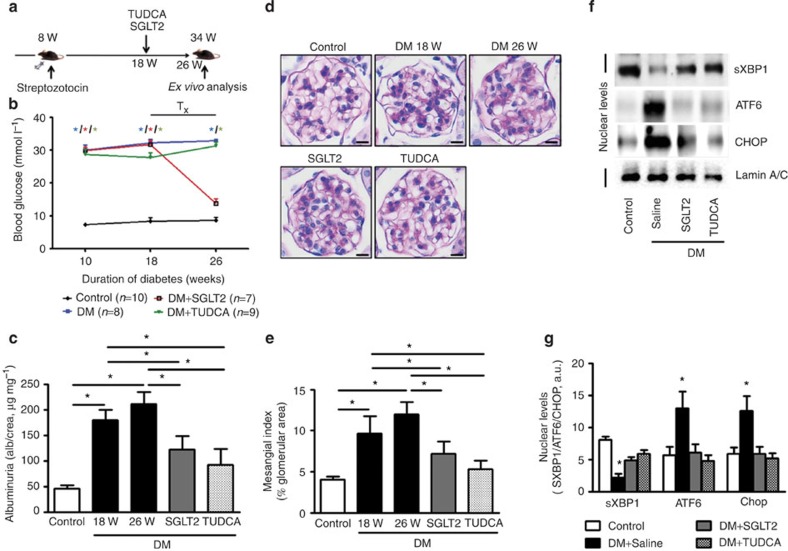
Hyperglycaemia-induced ER-stress response is causally linked to DN. (**a**,**b**) Therapeutic interventions with a SGLT2 inhibitor (dapagliflozin) normalizes blood glucose levels in mice: (**a**) schematic illustration of interventional studies in mice with STZ-induced hyperglycaemia. Treatment with a sodium–glucose co-transporter 2 (SGLT2) inhibitor or the chemical chaperone tauroursodeoxycholic acid (TUDCA) was initiated after manifestation of albuminuria at week 18. (**b**) Line graphs reflecting blood glucose levels (mean±s.e.m.) in mice with STZ-induced hyperglycaemia. Blood glucose was measured at indicated time points. Treatment with the SGLT2 inhibitor dapagliflozin normalizes blood glucose levels, while TUDCA has no impact on the blood glucose levels. T_x_: treatment period. (**c**–**g**) Normalizing glucose levels or attenuation of ER stress reduces albuminuria (**c**) and extracellular matrix deposition (**d**,**e**) and normalizes nuclear levels of sXBP1, ATF6 and CHOP (**f**,**g**);. C=control mice without diabetes, open bars; DM=diabetic mice, black bars; SGLT2 inhibitor: dapagliflozin treatment, grey bars; TUDCA treatment, dotted bars; scale bar represents (**c**: 20 μm); Mean±s.e.m.: *P<0.05,**P<0.01 (**b**,**c**,**e**,**g**: ANOVA).

To ascertain the causal role of hyperglycaemia-induced ER stress *in vivo*, we employed a sodium–glucose co-transporter 2 inhibitor (SGLT2 inhibitor-dapagliflozin) to normalize blood glucose levels^26^ and the chemical chaperone TUDCA to attenuate ER stress^27^. Lowering blood glucose levels with the SGLT2 inhibitor or treatment with TUDCA normalized nuclear levels of sXBP1, ATF6 (50 kDa) and CHOP and reduced albuminuria and indices of DN (Fig. 3), demonstrating that the hyperglycaemia-induced maladaptive UPR is causally linked to DN. Of note, these effects were apparent despite initiation of treatment 18 weeks after stable induction of hyperglycaemia and disease onset, reflecting partial disease reversal (Fig. 3).

**Figure 4:**
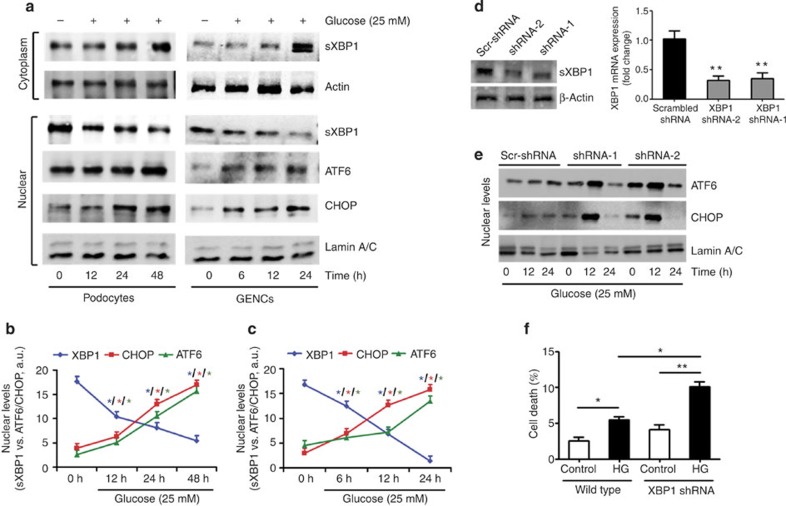
XBP1 limits hyperglycaemia-induced ER-stress response in glomerular cells. (**a**–**c**) Representative immunoblots showing cytoplasmic (**a**, top panel) and nuclear levels (**a**, lower panel) of ER transcription factors in immortalized mouse podocytes (left panel) and human glomerular endothelial cells (right panel) at indicated time points after treatment with high glucose (25 mM). Line graphs (**b**, podocytes and **c**, glomerular endothelial cells) reflecting the mean±s.e.m. of six independent experiments. (**d**–**f**) Representative immunoblot showing shRNA-mediated knockdown of XBP1 in immortalized mouse podocytes (**d**, left panel). Results for two independent shRNAs (shRNA-1 and -2) and a non-specific scrambled-shRNA (Scr-shRNA) are shown. Bar graph showing reduction of XBP1 expression in XBP1^KD^ cell lines, as determined by qRT–PCR (**d**, right panel). Representative immunoblots showing nuclear levels of cleaved ATF6 and CHOP in immortalized mouse control (Scr-shRNA) and XBP1 knockdown (shRNA-1 and -2) podocytes at indicated time points after treatment with high glucose (25 mM, **e**). Bar graph summarizing frequency (mean±s.e.m. of three independent experiments) of apoptotic cells as determined by TUNEL in immortalized mouse control and XBP1 knockdown podocytes 24 h after treatment with high glucose (25 mM, **f**). GENCs (glomerular endothelial cells); Mean±s.e.m. (**b**–**d**,**f**); *P<0.05, **P<0.01 (**b**–**d**: ANOVA; **f**: *t*-test).

To determine whether hyperglycaemia is sufficient to cell-autonomously initiate the maladaptive UPR, we determined sXBP1, ATF6 and CHOP levels in glucose-treated podocytes and glomerular endothelial cells (GENCs) *in vitro*. In both cell types high glucose but not mannitol-impaired nuclear translocation of sXBP1 associated with increased ATF6 (50 kDa) and CHOP levels (Fig. 4a–c and Supplementary Fig. 4). These data demonstrate that, unlike pharmacological ER-stress inducers such as tunicamycin or thapsigargin, which can activate all three major pathways of the UPR, hyperglycaemia selectively impairs nuclear translocation of sXBP1 while activating the ATF6 and CHOP branches of the UPR in DN.

This raises the question as to whether derangement of a single UPR branch is of primary mechanistic relevance or whether the branches are regulated independently, each contributing to DN. IRE1-XBP1 is the most conserved branch of UPR, and is the only branch of UPR present in lower eukaryotes regulating both protein folding and degradation^28^,^29^. Activation and nuclear translocation of spliced-XBP1 has been linked with cytoprotective effects^14^,^17^,^18^. To determine whether loss of XBP1 is sufficient to induce the observed pattern of the UPR response, we knocked down expression of XBP1 in podocytes (XBP1^KD^) using lentiviral shRNA (Fig. 4d). Indeed, reduced XBP1 expression markedly aggravated induction of nuclear ATF6 (50 kDa) and CHOP and increased cell death in glucose-stressed podocytes (Fig. 4e,f). These data identify a crucial function of XBP1 for restraining the glucose-induced maladaptive ER response.

### Podocyte-specific deletion of XBP1 promotes ER stress in DN

**Figure 5:**
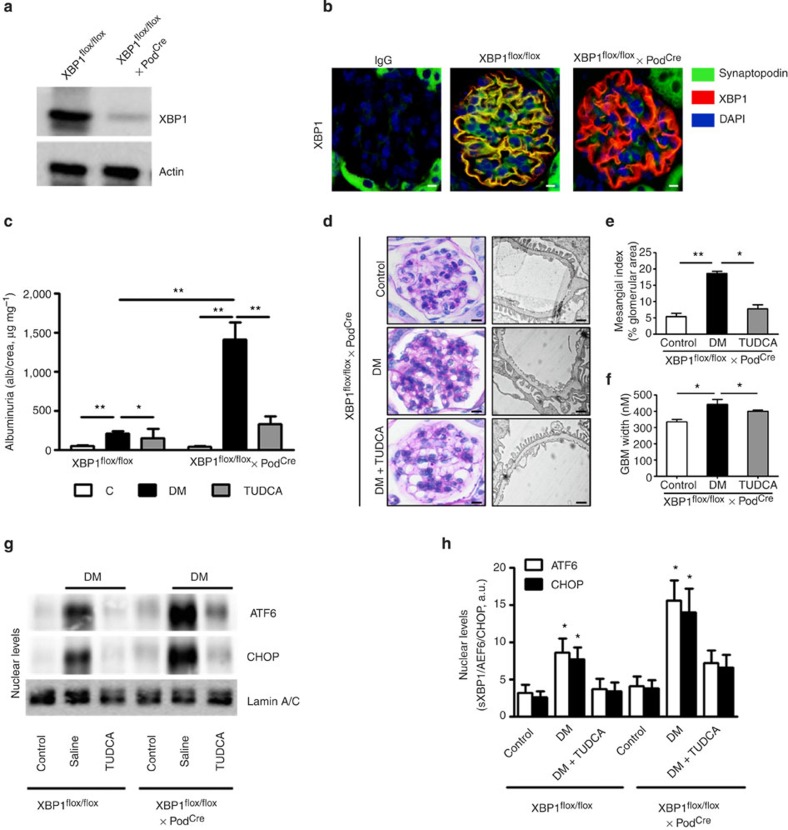
Podocyte-specific loss of XBP1 promotes maladaptive UPR in DN. Analyses of mice with podocyte-specific loss of XBP1 (XBP1^flox/flox^ x Pod^Cre^) compared with control mice (XBP1^flox/flox^). (**a**) Representative immunoblot images showing podocyte-specific nearly complete deletion of XBP1 in podocytes isolated from XBP1^flox/flox^ x Pod^Cre^ mice when compared with podocytes isolated from control mice (Xbp1^flox/flox^). (**b**) Exemplary images showing podocyte-specific depletion of XBP1 in glomeruli of wild-type (XBP1^flox/flox^) mice and XBP1^flox/flox^ crossed with Pod^Cre.^ (**c**–**h**) Analyses of DN in mice with podocyte-specific loss of XBP1. Bar graph summarizing albuminuria (**c**), representative images of the extracellular matrix deposition (**d**, left panel, PAS staining) and the glomerular filtration barrier (**d**, right panel, transmission electron microscopy, TEM), bar graph reflecting extracellular matrix deposition (**e**), glomerular basement membrane thickness (**f**), representative immunoblots (**g**) and bar graph (**h**) showing nuclear levels of ER transcription factors in renal cortex samples; (*n=*8 mice per group were analysed; for TEM (**d**) *n=*3 mice per group were analysed). C=control mice without diabetes, open bars; DM=diabetes, black bars; scale bar represents (**d**: 20 μm for PAS stain, 2 μm for glomerular filtration barrier, TEM); Mean±s.e.m. (**c**,**e**–**h**), *P<0.05, **P<0.01 (**c**–**g**: ANOVA; **h**: *t*-test).

The above data demonstrate that nuclear translocation of sXBP1 is impaired in diabetic mice and glucose-treated cells, resulting in enhanced activation and nuclear translocation of ATF6 (50 kDa) and subsequently CHOP levels. To ascertain the pathophysiological relevance of XBP1 for DN, we induced persistent hyperglycaemia in mice lacking XBP1 specifically in podocytes (XBP1^flox/flox^ x Pod^Cre^) (Fig. 5a,b). Markers of DN, including albuminuria, glomerular basement membrane (GBM)-width, and nuclear ATF6 and CHOP levels were markedly induced in XBP1^flox/flox^ x Pod^Cre^ mice when compared with wild-type mice (Fig. 5c–h). Moreover, we investigated whether inhibition of ER stress using the chemical chaperone TUDCA normalizes features of DN. Remarkably, treatment with TUDCA for a period of 6 weeks (as described in Fig. 3a) significantly reversed ER stress and features of DN (Fig. 5c–h). These results establish that loss of XBP1 expression in podocytes is sufficient for development of hyperglycaemia-induced ER stress in DN.

### Podocyte-specific ATF6 induction augments DN

**Figure 6:**
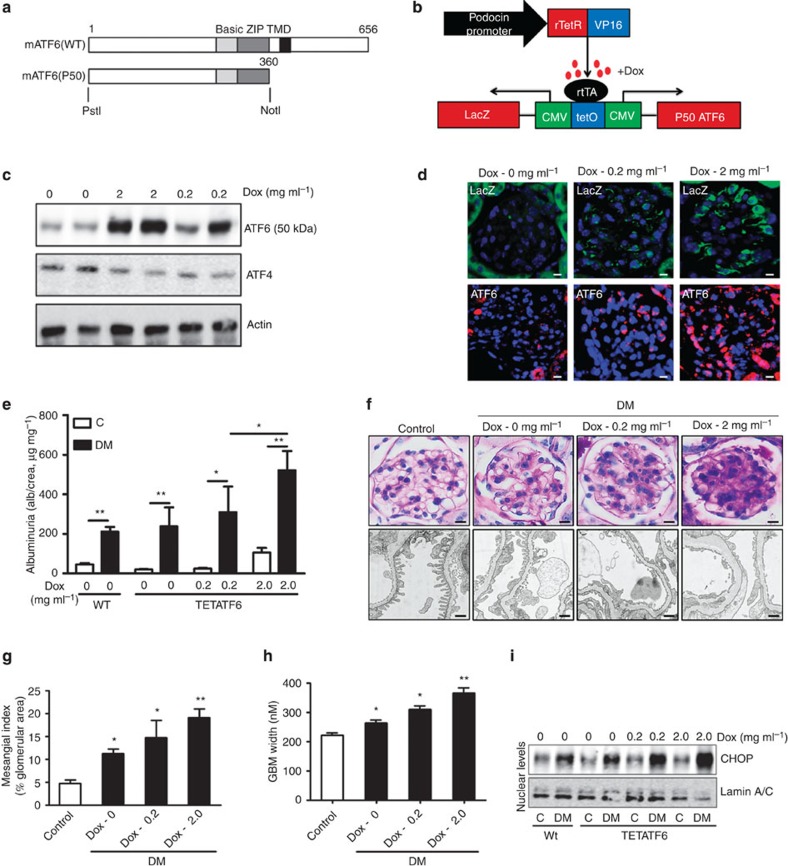
Podocyte-specific ATF6 induction exacerbates DN. Analyses of mice with podocyte-specific doxycycline (Dox)-inducible expression of p50-ATF6. (**a**) Structure of mouse ATF6 consisting of 656 amino acids. The positions of the basic region (Basic), the leucine zipper (ZIP) and the transmembrane domain (TMD) are indicated. The P50 ATF6 mutant (1–360) contains the basic region and leucine zipper. (**b**) Schematic illustration of mice model (TETATF6) used (**c**) Representative immunoblots showing induced expression of ATF6 (50 kDa) but not ATF4 in TETATF6-untreated mice and mice treated with doxycycline. (**d**) Representative images showing enhanced expression of beta galactosidase (lacZ, Top panel) and p50-ATF6 (lower panel) in TETATF6-untreated mice and mice treated with doxycycline (Dox=doxycycline at indicated concentrations within the drinking water). (**e**) Bar graph summarizing albuminuria, (**f**) representative images of the extracellular matrix deposition (**f**, upper panel, PAS staining) and the glomerular filtration barrier (**f**, lower panel, TEM), bar graph reflecting extracellular matrix deposition (**g**) and glomerular basement membrane thickness (**h**); (**i**) representative immunoblots showing nuclear levels of CHOP in wild type and doxycycline-treated mice. (*n=*6 mice per group were analysed). For TEM (**f**, lower panel) *n=*3 mice per group were analysed. C=control mice without diabetes, open bars; DM: diabetes, black bars; TETATF6: mice with tetracycline inducible podocyte-specific ATF6 expression. Scale bar represents (**d**: 20 μm and **f**: 20 μm for PAS stain, 2 μm for glomerular filtration barrier, TEM); mean±s.e.m. (**e**,**g**,**h**; ANOVA), *P<0.05, **P<0.01.

Loss of XBP1 induces ATF6 and CHOP, the latter being a potential converging point of a maladaptive, pro-apoptotic UPR response^10^,^30^. As ATF4 remained unchanged in DN, we speculated that ATF6 mediates transcriptional activation of CHOP in DN^31^,^32^. Indeed, electrophoretic mobility shift assay (EMSA) from nuclear extracts of renal cortex samples showed an increase in binding of p50 ATF6 to the ER-stress response element (ERSE2) of the CHOP promoter in DN (Supplementary Fig. 5). To explore the role of ATF6 activation in DN, we generated a bitransgenic mouse model with podocyte-specific, inducible expression of p50 ATF6 together with *LacZ*-encoding *β*-galactosidase (Fig. 6a,b and Supplementary Fig. 6). Podocyte-specific inducible expression of the transgenes on doxycycline treatment was confirmed by analysing both *β*-galactosidase and ATF6 (Tet-ATF6 mice, Fig. 6c,d)^33^. Doxycycline-induced ATF6 expression dose dependently exacerbated albuminuria, extracellular matrix deposition, GBM-width and CHOP expression (Fig. 6e–i). Considering the protection from DN in CHOP-null mice^34^, our data demonstrate that ATF6-dependent CHOP activation is sufficient to aggravate DN. Taken together, these results demonstrate that loss of XBP1 and induction of ATF6 in podocytes are sufficient for a maladaptive UPR *in vivo*, which is causally linked to DN.

### The glomerular p85-sXBP1 pathway is impaired in DN

Physiologically, UPR is a tightly controlled cellular stress response. However, the contribution of metabolic alterations in diabetes such as reduced insulin levels and/or impaired insulin signalling to the deranged UPR, thereby propagating a maladaptive ER response, particularly in podocytes remains unknown. It is well established that insulin signals in podocytes via the insulin receptor (INSR)^35^,^36^,^37^ but the pathophysiological relevance of insulin signalling in DN, and the intracellular molecular mechanisms involved, are hitherto unknown^38^. In hepatocytes insulin regulates nuclear translocation of sXBP1 via the regulatory subunits of PI3Kinase p85α and p85β. Disruption of the interaction between p85α and p85β with sXBP1 impairs insulin-induced nuclear translocation of sXBP1, which is causally linked to insulin resistance in obesity^17^,^39^.

**Figure 7:**
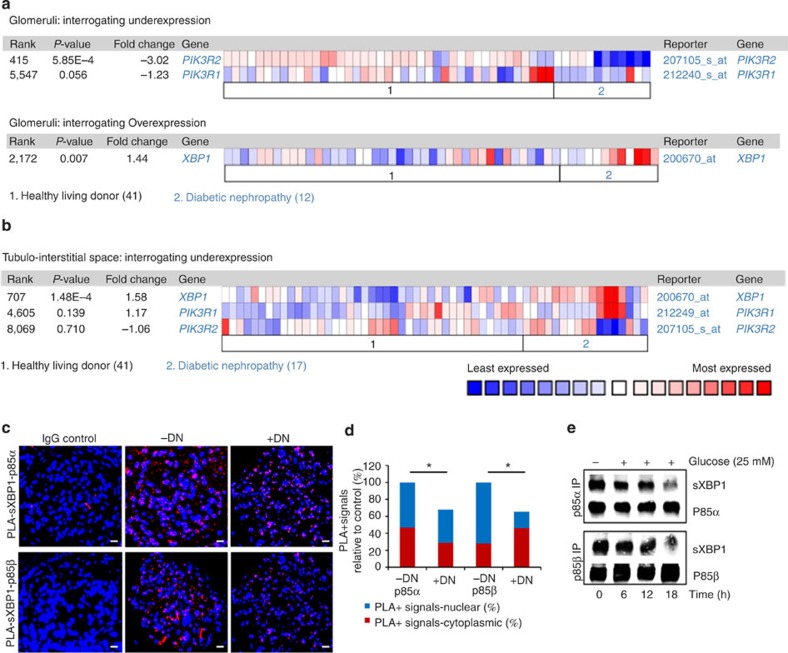
Impaired sXBP1–p85 interaction in human and murine DN. (**a**) Heat map obtained from ‘Ju Podocyte’ group from Nephromine, showing the glomerular-specific gene expression levels of p85α (PIK3R1), p85β (PIK3R2) and XBP1 in human healthy living donors (*n=*41) and patients with diabetic nephropathy (*n=*12). *P* values shown reflect results if interrogating the Nephromine database for underexpression (PIK3R1 and PIK3R2) or overexpression (XBP1). (**b**) Heat map obtained from ‘Ju Podocyte’ group from Nephromine, showing tubulointerstitium-specific gene expression levels of XBP1, p85α (PIK3R1), and p85β (PIK3R2) in human healthy living donors (*n=*41) and patients with diabetic nephropathy (*n=*17). *P* values shown reflect results if interrogating the Nephromine database for overexpression. (**c**,**d**) Representative images showing protein complexes (red) of sXBP1-p85α (**c**, upper panel) and sXBP1-p85β (**c**, lower panel) analysed by proximity-ligation assay (PLA) in renal biopsies obtained from diabetic humans without (–DN) or with (+DN) diabetic nephropathy. Frequency of nuclear and cytoplasmic protein complexes of five samples per group were summarized as bar graphs (**d**). Scale bar represents (**c**: 20 μm); mean±s.e.m. (**d**), *P<0.05, **P<0.01 (ANOVA). (**e**) Representative immunoblots of immunoprecipitates showing binding of sXBP1 with p85α and p85β in immortalized human podocytes after treatment with high glucose (HG 25 mM) at indicated time points. Representative immunoblots of three independent repeat experiments are shown.

To determine the pathophysiological relevance of the INSR—p85 pathway in DN, we first analysed p85α (PIK3R1) and p85β (PIK3R2) gene expression (mRNA levels) in a large human renal database (Nephromine)^23^,^24^. We analysed the gene expression (loss of function—under expression) in ‘Ju Podocyte’ database. In agreement with the above *in vivo* and *in vitro* observations, gene expression of p85α (PIK3R1) is slightly decreased (*P*=0.056, *t*-test) and that of p85β (PIK3R2) markedly (*P<*0.001, *t*-test) reduced (Fig. 7a). Intriguingly, reduced p85α and p85β gene expression was only observed in renal glomeruli but not in the tubulointerstitial compartment (Fig. 7b). In contrast, when interrogating the glomerular-specific overexpression, total-XBP1 mRNA levels were found to be upregulated in patients with DN when compared with healthy controls (*P=*0.007, *t*-test) (Fig. 7a, lower panel), similar to our observation made in db/db mice (Supplementary Fig. 1f). Importantly, the data obtained from nephromine reflect total XBP1 mRNA levels and preclude any conclusions regarding XBP1 splicing or nuclear translocation of sXBP1. To analyse mRNA levels of total-XBP1 and sXBP1, we performed quantitative qRT–PCR in renal cortex samples from murine DN. These data show that total-XBP1 and sXBP1 expression was not significantly altered in STZ-treated mice (26 weeks post-STZ) when compared with non-diabetic control mice (Supplementary Fig. 7). These data demonstrate that sXBP1 nuclear translocation is impaired independent of its total expression in DN (Fig. 1 and Fig. 2a–d).

Low levels of nuclear sXBP1 in DN despite sustained sXBP1 levels may be accounted for by an impaired interaction of p85 with sXBP1 (ref. 17). Hence, to ascertain whether the p85–XBP1 interaction is impaired in human DN we performed proximity ligation assay (PLA) to detect *in situ* protein–protein interactions of p85α-sXBP1 and p85β-sXBP1 in human renal biopsies. This revealed a marked reduction of both p85α-sXBP1 and p85β-sXBP1 protein complexes in renal biopsies from DN patients when compared with diabetic patients without DN (Fig. 7c,d). These observations prompted us to further investigate whether hyperglycaemia is sufficient to impair p85α and p85β interaction with sXBP1 in human podocytes. Treatment of human podocytes with high glucose reduced binding of p85α and p85β with sXBP1 in a time-dependent manner (Fig. 7e). These data suggest that signalling via the INSR-p85 pathway regulates ER-stress response and DN.

### Insulin signalling via p85α/p85β promotes adaptive UPR in DN

**Figure 8:**
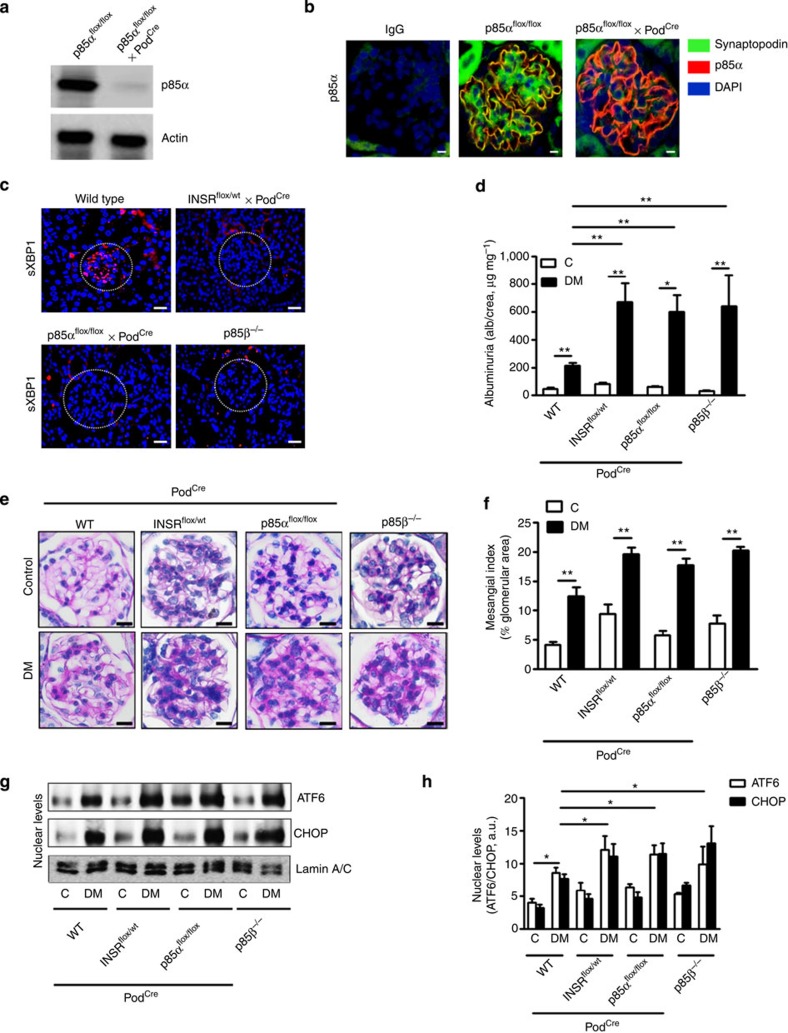
Signalling via INSR–p85 in podocytes is required for the adaptive ER-stress response in DN. (**a**) Representative image showing immunoblot analysis of podocyte-specific nearly complete deletion of XBP1 and p85α in podocytes isolated from p85α^flox/flox^ x Pod^Cre^ mice when compared with podocytes isolated from control mice (p85α^flox/flox^ mice). (**b**) Exemplary images showing podocyte-specific depletion of p85α in renal glomeruli of wild-type (p85α^flox/flox^) mice and p85α^flox/flox^ mice crossed with Pod^Cre^. (**c**) Representative images showing sXBP1 levels (using an antibody detecting the spliced form of XBP1) in the renal glomeruli of non-diabetic wild-type mice and mice with podocyte-specific deletion of insulin receptor (INSR^flox/Wt^ x Pod^Cre^) or p85α (p85α^flox/flox^ x Pod^Cre^) or p85β deficiency (p85β^−/−^). (**d**–**h**) Bar graph summarizing albuminuria (**d**), representative images of the extracellular matrix deposition (**e**) bar graph reflecting extracellular matrix deposition (**f**), representative immunoblots (**g**), and bar graph (**h**) showing nuclear levels of ER transcription factors in renal cortex samples; (*n=*8 mice per group were analysed). C=control mice without diabetes, open bars; DM=diabetes, black bars. Mean±s.e.m. (**d**,**f**,**h**), *P<0.05, **P<0.01 (ANOVA).

To evaluate the pathophysiological role of INSR signalling, we generated mice with podocyte-specific deletion of the INSR. In agreement with previous reports^36^, mice with homozygous ablation of INSR in podocytes (INSR^flox/flox^ x Pod^Cre^) developed mild albuminuria at age 8 weeks even in the absence of hyperglycaemia (Supplementary Fig. 8a-b). Hence, we used mice with podocyte-specific INSR heterozygosity (INSR^flox/Wt^ x Pod^Cre^), which lack spontaneous proteinuria. Consistent with the above data obtained from human biopsies, we observed nuclear-localized sXBP1 predominantly in glomeruli of non-diabetic wild-type mice, whereas nuclear sXBP1 was absent in mice with defective INSR signalling in podocytes (Fig. 8c and Supplementary Fig. 8c). In addition, insulin promotes nuclear translocation of sXBP1 in podocytes *in vitro*, indicating that insulin physiologically regulates XBP1 activity in podocytes (Supplementary Fig. 8d). Indeed, features of DN were exacerbated in diabetic INSR^flox/Wt^ x Pod^Cre^ mice, confirming a crucial role of insulin signalling for podocyte function in DN (Fig. 8d–h). To investigate whether insulin-induced nuclear translocation of sXBP1 depends on its canonical activation pathway regulated by IRE1α phosphorylation, we treated podocytes with insulin. Insulin (100 nM), which was sufficient to promote nuclear translocation of sXBP1, failed to induce IRE1α phosphorylation (Supplementary Fig. 8d), indicating that insulin-mediated sXBP1 nuclear translocation is independent of IRE1α activation and rather depends on transactivation of the downstream signalling intermediates of INSR, the PI3K subunits p85α and p85β. To formally demonstrate this hypothesis, we genetically inactivated the downstream signalling intermediates of the INSR, p85α (p85α^flox/flox^ x Pod^Cre^) and p85β (p85β^−/−^) *in vivo* (Fig. 8a,b). Again, features of DN were markedly aggravated in diabetic p85α^flox/flox^ x Pod^Cre^ and p85β^−/−^ mice (Fig. 8d–h). Importantly, nuclear translocation of sXBP1 was impaired while nuclear ATF6 (50 kDa) and CHOP were increased in diabetic INSR^flox/Wt^ x Pod^Cre^, p85α^flox/flox^ x Pod^Cre^ and p85β^−/−^ mice, demonstrating that insulin signalling via INSR-p85-sXBP1 axis is indispensable for the adaptive ER-stress response in DN (Fig. 8d–h).

## Discussion

**Figure 9:**
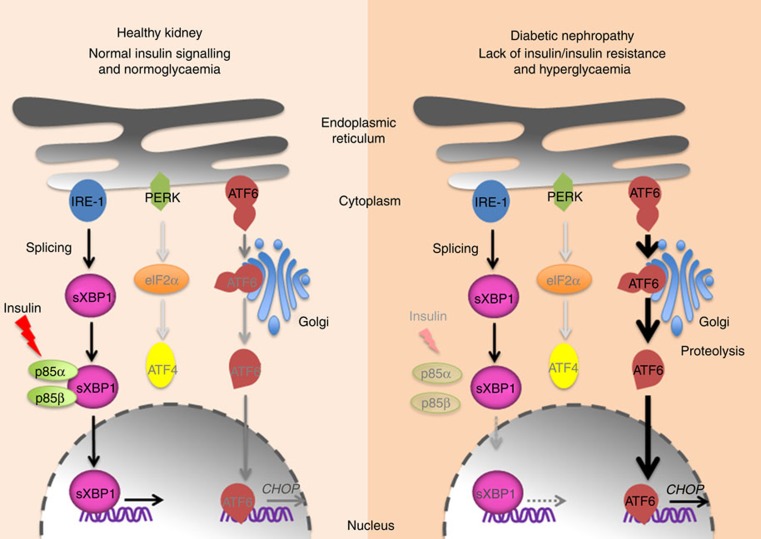
Proposed model: disparate regulation of the UPR in DN. In healthy subjects insulin signalling induces nuclear translocation of sXBP1 by promoting its interaction with regulatory subunits of PI3Kinase p85α and p85β. In contrast, in diabetic individuals lack of insulin (type 1 diabetes mellitus) or impaired insulin signalling (type 2 diabetes mellitus) in combination with hyperglycaemia selectively impairs p85-dependent sXBP1 nuclear translocation, impairing adaptive XBP1 signalling and resulting in maladaptive ER-stress signalling via activation and nuclear translocation of ATF6 and CHOP.

The comprehensive analysis of the three principle UPR response arms in the current study, including analyses of nuclear levels of ATF6, ATF4 and sXBP1, unveils a previously unrecognized role of insulin and the UPR in DN. These data establish that a maladaptive ER response, which is characterized by a disparate regulation of the tripartite-UPR, is causally linked to glomerular cell dysfunction and DN. Signalling via the XBP1 branch of the UPR is required for an adaptive ER response in DN, while genetic disruption or functional inactivation of this pathway in murine models of type 1 diabetes mellitus (lack of insulin) and type 2 diabetes mellitus (impaired insulin signalling secondary to insulin resistance) promotes a maladaptive UPR. The latter is hallmarked by impaired sXBP1 nuclear translocation, which provokes a maladaptive ER response characterized by ATF6 and CHOP signalling in DN (Fig. 9). These results shed new light onto the regulation of the UPR in DN and identify a new function of insulin signalling in podocytes.

The high levels of nuclear sXBP1 at baseline in renal cortex extracts, podocytes and GENCs concur with previous studies demonstrating that sXBP1 is required in a tissue-specific manner for a constitutive-homeostatic UPR (in contrast to the inducible UPR in disease conditions)^11^,^12^,^13^,^14^. The function of sXBP1 for a constitutive UPR has been established in secretory and non-secretory cells and is essential to maintain tissue homeostasis by controlling the transcription of a core group of genes—in a tissue and context specific manner—involved in constitutive maintenance of ER function in various cell types^11^,^12^,^13^,^14^,^40^. For example, deletion of XBP1 in intestinal epithelial cells results in spontaneous enteritis and increased susceptibility of experimental colitis, reflecting the crucial role of constitutive XBP1 expression in intestinal epithelial cells^14^. Our observations add to these findings, as they describe for the first time the requirement of XBP1 for maintaining homeostasis in glucose-stressed renal cells (podocytes). On the basis of the current observation, we speculate that the kidney, including the terminally differentiated podocytes, is continuously exposed to stressors, such as fluid pressure and rapid changes in the extracellular milieu. Furthermore, the fractional rate of protein synthesis by the kidney has been shown to approximate 42% of the total daily body load^41^. Thus, like intestinal epithelial cells^14^ renal cells appear to be exposed to a stressful environment, necessitating a constitutive, XBP1-dependent UPR to maintain tissue homeostasis. Disruption of the XBP1-mediated constitutive UPR in podocytes predisposes the kidney to glucose-induced cellular dysfunction and injury. Importantly, this constitutive UPR depends on insulin signalling, and disturbance of the INSR/P85/XBP1-mediated constitutive UPR in podocytes, for example, in type 1 diabetes (insulin deficiency) or type 2 diabetes (insulin resistance), is mechanistically linked with glomerular disease.

A limitation of our study is that p85β^LoxP/LoxP^ mice were not available to us and hence we were not able to inactivate p85β specifically in podocytes. While the effects in other cell types may obscure the observations in mice with complete p85β deletion, the results obtained in these mice are in alignment with those in mice with podocyte-specific deletion of INSR, p85α or XBP1. Furthermore, we have not entirely clarified how sXBP1 nuclear deficiency provokes ATF6 activation and nuclear translocation. Transcriptional induction of mammalian ER-quality control proteins is mediated by single or combined actions of ATF6 and XBP1. ATF6 can heterodimerize with XBP1 and the resulting heterodimer gains broader DNA-binding specificity for induction of ERAD components^42^. Considering sXBP1 and ATF6 both bind to the ERSE2 site within the CHOP promoter^43^, sXBP1 may act as a repressor in podocytes. Future studies investigating XBP1-dependent regulation of ATF6 activity are required to gain more mechanistic insights into the regulation of the constitutive and inducible UPR in podocytes and other renal cells. This may identify new therapeutic targets for DN.

The link between diabetes and CKD is well established. In addition, a number of studies demonstrated an association between the metabolic syndrome (which is typically associated with impaired insulin sensitivity) or insulin resistance itself and an increased risk for CKD^44^. Several mechanisms, such as increased reactive oxygen species, inflammation, or altered adipokine levels, have been proposed^45^,^46^,^47^, but the exact mechanism resulting in CKD in insulin resistance remained elusive hitherto. Our study, using a combination of two independent mouse models, *in vitro* studies and human renal biopsy analyses establish a direct mechanistic link between defective insulin signalling in podocytes and impaired kidney function.

A role for insulin signalling in regulation of podocyte function has been previously proposed, but the pathophysiological relevance and its specific intracellular molecular targets in a CKD, particularly in DN, has not been shown so far. By linking impaired insulin signalling in podocytes with the induction of a maladaptive ER-stress response in DN, these results provide a unifying mechanism that contributes to progression of DN in both type 1 and type 2 diabetes and potentially even in insulin-resistant individuals without overt diabetes.

Whether impaired insulin/XBP1 signalling in podocytes is always associated with insulin resistance in tissues like the liver, skeletal muscles or adipose tissues remains to be evaluated. Alternatively, insulin resistance in podocytes may occur independently of other tissues and/or the INSR/PI3K/XBP1 pathway may be selectively impaired in podocytes, reflecting cell or pathway selective insulin resistance^48^. Of note, in the current study, we show that TUDCA, which has been shown to improve insulin sensitivity in mice and humans^49^, ameliorated indices of DN in mice with genetic ablation of the XBP1, which is a crucial downstream regulator of INSR/p85 signalling. Currently, it is not known if TUDCA treatment would likewise have beneficial effects in human patients with DN. On the basis of our *in vivo* studies in animal models, we propose that treatment of patients with DN with TUDCA may be a feasible approach to DN, which should be evaluated in clinical studies.

In addition to the bile acid derivative TUDCA, inhibition of SGLT2 ameliorated markers of DN in mice. Initial concerns that the increased intratubular glucose levels following SGLT2 inhibition may cause tubular damage in diabetic patients have recently been largely precluded, at least in animal and *in vitro* studies^50^. Rather, SGLT2 inhibition turned out to be tubulo-protective, presumably by preventing glucose uptake, and thus intracellular hyperglycaemia in tubular cells^50^. Our current finding establishes that normalization of blood glucose levels by SGLT2 inhibitors ameliorates not only tubular, but also glomerular changes, presumably by impeding the glucose-induced maladaptive UPR in the glomerulus. This provides additional and new evidence for a nephroprotective effect of SGLT2 inhibition.

Collectively, based on the current results we propose that impaired insulin signalling coupled with hyperglycaemia directly impedes sXBP1 activity in podocytes, thus exacerbating ATF6-dependent maladaptive ER response and DN. This pathway may enable the rationale design of new therapeutic approaches for DN.

## Methods

### Materials

The following antibodies were used in the current study: mouse monoclonal antibodies: sXBP1 (R&D biosystems), CHOP (New England Biolabs and Santacruz, Germany), ATF6 (Imgenex); rabbit polyclonal antibodies: XBP1, ATF4 and WT1; Goat polyclonal antibody: p85β (Santacruz, Heidelberg, Germany); rabbit polyclonal antibodies: p85α (Millipore GmbH, Germany); Lamin A/C (New England Biolabs, Germany), Beta galactosidase (Biorbyt Ltd, Cambridgeshire, United kingdom). The following HRP-conjugated secondary antibodies were used for immunoblotting: rabbit IgG (New England Biolabs, Germany), mouse IgG (Abcam). The following secondary antibodies for immunofluorescence were used: Texas red-conjugated anti-mouse and FITC-conjugated anti-mouse, FITC-conjugated anti-goat (Vector Laboratories, CA, USA).

The following reagents were obtained from Sigma-Aldrich, Taufkirchen, Germany: Duolink *In situ* proximity ligation assay kit and reagents, streptozotocin, RPMI 1640, 1% collagen, 2% Gelatin and Bradford reagent.

Other reagents used in the current study were Insulin (Lantus; Sanofi, Frankfurt, Germany); mouse albumin Elisa quantitation kit (Bethyl Laboratories, TX,USA); Trypsin-EDTA, fetal bovine serum and HEPES (PAA laboratories, Pasching, Austria); Interferon γ (cell sciences, Canton, MA); *In situ*-cell death detection kit (TUNEL) and protease inhibitor cocktail (Roche diagnostics GmbH, Mannheim, Germany); BCA reagent (Thermoscientific, Germany); Chemiluminescence EMSA kit (Active motif, La Hulpe, Belgium); Vectashield mounting medium with DAPI, (Vector Laboratories, CA, USA); shRNA for XBP1 (Openbiosystems, Heidelberg, Germany); transfection reagent FuGENE (Promega, Germany); and PVDF membrane and immobilion enhanced chemiluminescence reagent (Millipore GmbH, Germany).

### Mice

XBP1^flox/flox^ mice (provided by Laurie H. Glimcher), INSR^flox/flox^ mice (obtained from the The Jackson Laboratory) and PI3KR1^flox/flox^ (p85α) mice (obtained from The Jackson Laboratory) were crossed with Pod^Cre^ mice (provided by Marcus J. Moeller) to generate mice with podocyte-specific deletion of XBP1, INSR and p85α^17^,^18^,^36^. PIK3R2 (p85β)-constitutive-knockout mice were obtained from The Jackson Laboratory. Mice had been back crossed onto the C57BL/6 background for at least seven generations and were routinely maintained on the C57BL/6 background. Only littermates were used as controls. The presence of targeted genes and transgenes was routinely determined by PCR analyses of tail DNA, and podocyte-specific genetic deletion of respective genes was confirmed by two independent methods: (1) analysis of protein levels in isolated podocytes as compared with wild-type mice and (2) double immunofluorescence staining using a podocyte-specific marker (synaptopodin) and an antibody for the corresponding protein to be inactivated.

Podocyte-specific tetracycline-inducible ATF6-expressing mice were newly generated. We used the human podocin (NPHS2) gene promoter to control expression of the rtTA cassette (reverse tetracycline-controlled transcriptional activator)^33^ and bred these mice with a reporter mouse line generated by us, which contains the cytomegalovirus minimal promoter and tetO promoter elements together with the LacZ gene, encoding β-galactosidase and p50 ATF6. Administration of tetracycline in the drinking water induced podocyte-specific expression of β-galactosidase and increased ATF6 expression as determined by immunofluorescence staining. ATF6-inducible mice were backcrossed onto the C57BL/6 background for seven generations, and only littermates were used as controls in the current study.

Wild-type C57BL/6 and db/db (C57BL/KSJRj-db) mice were obtained from Janvier, France. Animal experiments were conducted following standards and procedures approved by the local Animal Care and Use Committee (Landesverwaltungsamt Halle, Germany).

### Induction of diabetes using streptozotocin

Diabetes was induced by intraperitoneal administration of streptozotocin (STZ) at 60 mg kg^−1^, freshly dissolved in 0.05 M sterile sodium citrate, pH 4.5, on five successive days in 8-week-old male mice. Mice were considered diabetic if blood glucose levels were above 300 mg dl^−1^ (16.7 mM l^−1^) 16–25 days after the last STZ injection. Blood glucose levels were determined in blood samples from the tail vein using ACCU-CHEK glucose sticks. In the first 3 weeks after onset of diabetes, blood glucose values were measured 3 times per week, afterwards once a week. Mice displaying blood glucose levels above 500 mg dl^−1^ (27.7 mM l^−1^) received 1–2 U insulin Lantus to avoid excessive and potentially lethal hyperglycaemia. We obtained blood and tissue samples after 26 weeks of persistent hyperglycaemia in diabetic mice. Age-matched littermates served as controls. We injected a subset of diabetic mice intraperitoneally with either TUDCA (150 mg kg^−1^, dissolved in saline) or saline once daily starting 18 weeks after the last streptozocin (STZ) injection until 1 day before analyses (week 26). In addition a group of diabetic mice received drinking water supplemented with the sodium-glucose co-transporter 2 (SGLT2) inhibitor (dapagliflozin, 25 mg kg^−1^, body weight, provided by Bristol-Myers Squibb) starting 18 weeks after the last streptozotocin (STZ) injection until 1 day before analyses (week 26).

### Determination of albuminuria

The day before blood sample collection and tissue preparation individual mice were placed in metabolic cages for 24 h and urine samples were collected. We determined urine albumin using a mouse albumin ELISA according to the manufacturer’s instructions and urine creatinine using a modified version of the Jaffe method using a commercially available assay (X-Pand automated platform, Siemens, Eschborn, Germany)^21^,^51^.

### Histology and immunohistochemistry

We perfused animals with ice-cold PBS and then with 4% buffered paraformaldehyde. Tissues were further fixed in 4% buffered paraformaldehyde for 2 days, embedded in paraffin and processed for sectioning. Extracellular matrix deposition in glomeruli was assessed by periodic acid–Schiff staining. An investigator scored the sections in a blinded manner, according to an established scoring system (range 0–3; 0: no extracellular matrix deposition; 3: extracellular matrix deposition in all sections of the glomeruli)^21^,^51^,^52^.

Human renal tissue samples were provided by the tissue bank of the National Center for Tumor Diseases (NCT, Heidelberg, Germany) in accordance with the regulations of the tissue bank and the approval of the ethics committee of the University of Heidelberg. Immunofluorescence analyses on human and mouse renal biopsies were essentially performed as previously described^53^. Double immunofluorescence of sXBP1, ATF6 and CHOP on paraffin sections was performed on human kidney sections with and without DN. In brief, sections were fixed in ice cold acetone for 1 min, incubated in PBS (0.1% triton+0.1% sodium citrate) for 10 min, blocked in 1.5% serum for 1 h and incubated overnight at 4 °C with the primary antibodies against human sXBP1 (1:50), ATF6 (1:50) and CHOP (1:50). Corresponding fluorescently labelled secondary antibodies (anti-mouse IgG-FITC: 1:200, anti-rabbit IgG-Texas red) were added for 60 min and sections were rinsed twice in PBS. Slides were covered with vectashield mounting medium containing nuclear stain DAPI. Specimens were analysed on a Leica SP5 confocal microscope. Nuclear localization of sXBP1, ATF6 and CHOP was analysed using NIH-ImageJ software. DAPI stained and fluorescently labelled images were acquired individually. The exposure settings and gain of laser were kept the same for each condition. Twenty fields were acquired per condition, a single focal plane by field. The ImageJ plugin, co-localization colour map was used for automatic quantification and visualization of co-localized fluorescent signals. Brightness and contrast was adjusted for all images to exclude background signal. Before beginning analysis, images were converted to 8-bit grey scale. ‘Co-localization’ was run to generate the Icorr values. The plugin is based on the method originally described elsewhere^52^,^54^.

### Reverse transcription–quantitative polymerase chain reaction

Mouse glomeruli from wild-type control non-diabetic and diabetic mice were isolated by the sequential sieving method^55^. Total RNA was isolated using RNeasy kit (Qiagen, Germany) and reverse-transcribed by using the RevertAid First Strand cDNA Synthesis Kit (Thermoscientific, Germany). Quantitative polymerase chain reaction was performed in a Bio-Rad real-time system (CFX-Connect) using SYBR Green (Thermoscientific, Germany). The mRNA levels of the genes tested were normalized to 18S as an internal control. The primer sequences were 18S, 5′- AGTCCCTGCCCTTTGTACACA and 5′- CGATCCGAGGGCCTCACTA -3′; DNAJB9, 5′- AGAGGCAATGGGAGTCCTTT -3′ and 5′- CCTGGAAGTGATGCCTTTGT -3′; Edem1, 5′- TCTCTCCTGGTGGAATTTGG -3′ and 5′- AATGGCCTGTCTGGATGTTC -3′; DNAJC3, 5′- CCGTGGAAGCCATTAGGATA -3′ and 5′- TAACCGCTGGGCTTTCTCTA -3′; PDIA4, 5′- GTGGTCATCATTGGGCTCTT -3′ and 5′- CTTCTCAGGGTGTGTCAGCA -3′; Ero1B, 5′- CAGCAAACAGCACCAAAGAA -3′ and 5′- TGGTCCTGCGAATCATCATA -3′; XBP1, 5′- GGTCTGCTGAGTCCGCAGCAGG -3′ and 5′- AGGCTTGGTGTATACATGG -3′; sXBP1, 5′- GGTCTGCTGAGTCCGCAGCAGG -3′ and 5′- GAAAGGGAGGCTGGTAAGGAAC -3′; ATF4, 5′- CATGCCAGATGAGCTCTTGA -3′ and 5′- GCCAATTGGGTTCACTGTCT -3′.

### Transmission electron microscopy

Transmission electron microscopy (TEM) was performed at the Institute for Clinical Chemistry and Pathobiochemistry, Otto-von-Guericke-University Magdeburg and at the Institute of Anatomy, University Leipzig, Germany. Renal cortex tissues were fixed with 2.5% glutaraldehyde, 2.5% polyvidone 25, 0.1 M sodium cacodylate pH 7.4. After washing with 0.1 M sodium cacodylate buffer (pH 7.4), samples were post-fixed in the same buffer containing 2% osmium tetroxide and 1.5% potassium ferrocyanide for 1 h, washed in water, contrasted *en bloc* with uranyl acetate, dehydrated using an ascending series of ethanol and embedded in glycidyl ether 100-based resin. Ultrathin sections were cut with a Reichert Ultracut S ultramicrotome (Leica Microsystems, Wetzlar, Germany), contrasted with uranyl acetate and lead citrate and were viewed with an EM 10 CR electron microscope (Carl Zeiss NTS, Oberkochen, Germany). The GBM thickness was analysed by NIH-ImageJ software^56^.

### *In situ* proximity ligation assay

A Duolink *in situ* PLA Kit was used according to the manufacturer’s instructions (Olink Biosciences, Sigma Aldrich) for *in situ* proximity-ligation assay. Double immunofluorescence staining with primary antibodies was performed as described above, followed by PLA to detect the sXBP1 and p85 protein complexes on paraffin sections of human renal biopsies. For quantification of PLA data, images were acquired on a Olympus microscope (BX43, Germany). The exposure settings and gain of laser were kept the same for each condition. Thirty fields were acquired per condition, a single focal plane by field. Before beginning analysis, images were converted to 8-bit images on ImageJ-Fiji. A threshold range was set to distinguish the objects of interest from the background. Automated particle analysis was used to detect the nuclei count per glomeruli. To exclude ‘noise’, the size of particles was defined between 150-Infinity pixels^2 and roundness values were limited to 0.00–1.00. The accumulative counts for each cell type appeared in the Counters menu. After counting the cells within the ROI, the size of the ROI was calculated and used to identify the number of cells labelled per glomeruli. To count the PLA-positive signals, *Point Picker* plugin was used. The Point List dialog enabled the calculation of PLA-positive signals in the nucleus and cytoplasm.

### Cell culture

Conditionally immortalized mouse podocytes (obtained from Jochen Reiser’s laboratory, Rush University, Chicago) were cultured using well-established protocols^53^. Mouse podocytes were routinely grown on plates coated with collagen type 1 at 33 °C in the presence of interferon γ (10 U ml^−1^) to enhance expression of a thermosensitive T antigen. Under these conditions, cells proliferate and remain undifferentiated. To induce differentiation, podocytes were grown at 37 °C in the absence of interferon γ for 14 days. Experiments were performed after 14 days of differentiation. Differentiation was confirmed by determining expression of synaptopodin and Wilms tumour-1 protein. Immortalized mouse GENCs were grown in RPMI 1640 medium with 10% heat-inactivated fetal calf serum^53^. Cells were starved overnight before treatment with high concentrations of glucose (25 mM) or mannitol (25 mM). At desired time points post glucose and mannitol treatment, cytosolic and nuclear lysates were prepared for immunoblotting analysis.

### Determination of cell death by TUNEL assay

Podocytes were serum-starved overnight in serum-free medium followed by incubation with high concentrations of glucose (25 mM). At indicated time points post glucose treatment cells were fixed in 4% neutral buffered formalin, washed in PBS and apoptosis was determined using the TUNEL assay^53^. Cells were incubated with terminal deoxynucleotidyl transferase in the presence of fluorescein-labelled dUTP (60 min at 37 °C) and counterstained with Hoechst 33258 (3.5 μg ml^−1^). Random images were obtained and the frequency (in percent) of TUNEL-positive cells was determined by a blinded investigator.

### Production of lentiviral particles

VSV-G-pseudotyped lentiviral particles were generated as described^57^. In brief, HEK 293 T cells were transfected with pLKO.1-XBP1 together with the packaging plasmid psPAX2 (Addgene) and VSV-G-expressing plasmid (pMD2.g) (Addgene) using transfection reagent Fugene. Lentiviral particles in the supernatant were harvested at 36 h and 48 h post transfection. This lentiviral supernatant was added on the podocytes and knockdown efficiency was enumerated after 4 days by immunoblotting and quantitative RT–PCR.

### Cell fractionation

For isolation of cytosolic and nuclear fractions, renal cortex samples were lysed using tissue homogenizer in buffer-A containing 10 mM HEPES-KOH (pH-7.9), 10 mM KCL, 1.5 mM MgCl_2_, 1 mM EDTA, 0.6% NP-40, 0.5 mM DTT, protease inhibitor cocktail (Roche) and lysates were incubated for 10 min on ice. After brief vortexing the lysates were centrifuged for 30 s at 14,000 r.p.m. at 4 °C. Supernatants were collected as cytosolic fractions and the pellets were resuspended in 100 μl of buffer-B containing 10 mM HEPES-KOH (pH-7.9), 25% glycerol, 420 mM Nacl, 1.5 mM MgCl_2_, 0.2 mM EDTA, 0.5 mM DTT and protease inhibitors. Lysates were incubated for 20 min on ice followed by centrifugation at 13,000 *g* at 4 °C for 5 min. Supernatants containing the nuclear extracts were collected and stored at −80 °C. A similar procedure was used for isolation of cytosolic and nuclear fractions of cells where buffer-A contains 10 mM HEPES-KOH (pH-7.9), 10 mM KCL, 1.5 mM MgCl_2_, 0.5 mM DTT and protease inhibitors. Protein concentration was measured using Bradford reagent or BCA reagent, and purity of nuclear and cytoplasmic fractions was determined by lamin A/C and actin western blots, respectively.

### Immunoblotting

Cell lysates were prepared using RIPA buffer containing 50 mM Tris (pH7.4), 1% NP-40, 0.25% sodium-deoxycholate, 150 mM NaCl, 1 mM EDTA, 1 mM Na_3_VO_4_, 1 mM NaF supplemented with protease inhibitor cocktail. Lysates were centrifuged (13,000 *g* for 10 min at 4 °C) and insoluble debris was discarded. Protein concentration in supernatants was quantified using BCA reagent. Equal amounts of protein were electrophoretically separated on 10% or 12.5% SDS polyacrylamide gel, transferred to PVDF membranes and probed with desired primary antibodies at a concentration of XBP1 (1:200), ATF6 (1:000), CHOP (1:200), ATF4 (1:200), LaminA/C (1:1,000), Actin (1:1,000), p85α (1:1,000), p85β (1:200). After overnight incubation with respective primary antibodies at 4 °C, membranes were then washed with TBST and incubated with anti-mouse IgG (1:5,000), anti-rabbit IgG (1:2,000) or anti-Goat IgG (1:2,000) horseradish peroxidase-conjugated antibodies for 1 h at room temperature. Blots were developed with the enhanced chemiluminescence system. To compare and quantify levels of proteins, the density of each band was measured using Image J software. Equal loading for total cell or tissue lysates was determined by β-actin western blot. Uncropped scans of immunoblots are shown In Supplementary Figs 9,10.

### Electrophoretic mobility shift assay

Nuclear lysates from renal cortex samples were isolated as described above and subjected to chemiluminescent EMSA according to the manufacturer’s instructions (active motif). Following primers were used for the detection of ER-stress response element of the CHOP promoter: 5′- CCTACCAATCAGAAAGTGGCACGC -3′; 3′- GGATGGTTAGTCTTTCACCGTGCG -5′ (ref. 32).

### Gene expression analysis in Nephromine database

Gene expression data in healthy and DN patients were extracted from the Nephromine database (Life Technologies, Ann Arbor, MI, **Website:** **www.nephromine.org**). Within Nephromine data were obtained from ‘Ju Podocyte’ database, which is a collection of gene expression profiling of 217 micro-dissected glomerular samples and 219 tubulointerstitial samples from CKD patients, and healthy living donors was used to predict cell-type-specific (podocyte) transcripts by support vector machine-based *in silico* nano-dissection.

### Statistical analysis

The data are summarized as the mean±s.e.m. (standard error of the mean). Statistical analyses were performed with Student’s *t*-test or ANOVA as appropriate. *Post hoc* comparisons of ANOVA were corrected with the method of Tukey. StatistiXL software ( http://www.statistixl.com) and Prism 5 ( www.graphpad.com) software were used for statistical analyses. Statistical significance was accepted at values of *P*<0.05.

## Additional information

**How to cite this article:** Madhusudhan, T. *et al*. Defective podocyte insulin signalling through p85-XBP1 promotes ATF6-dependent maladaptive ER-stress response in diabetic nephropathy. *Nat. Commun.* 6:6496 doi: 10.1038/ncomms7496 (2015).

## Supplementary Material

Supplementary Information Supplementary Figures 1-10
