# Supplementary material for: Defective podocyte insulin signalling through p85-XBP1 promotes ATF6-dependent maladaptive ER-stress response in diabetic nephropathy
Source: Nat Commun. 2015 Mar 10;6:6496. doi: 10.1038/ncomms7496 (PMC4366504; doi:10.1038/ncomms7496)
Supplement: Supplementary Information — Supplementary Figures 1-10 [file ncomms7496-s1.pdf]

Supplementary Figure. 1

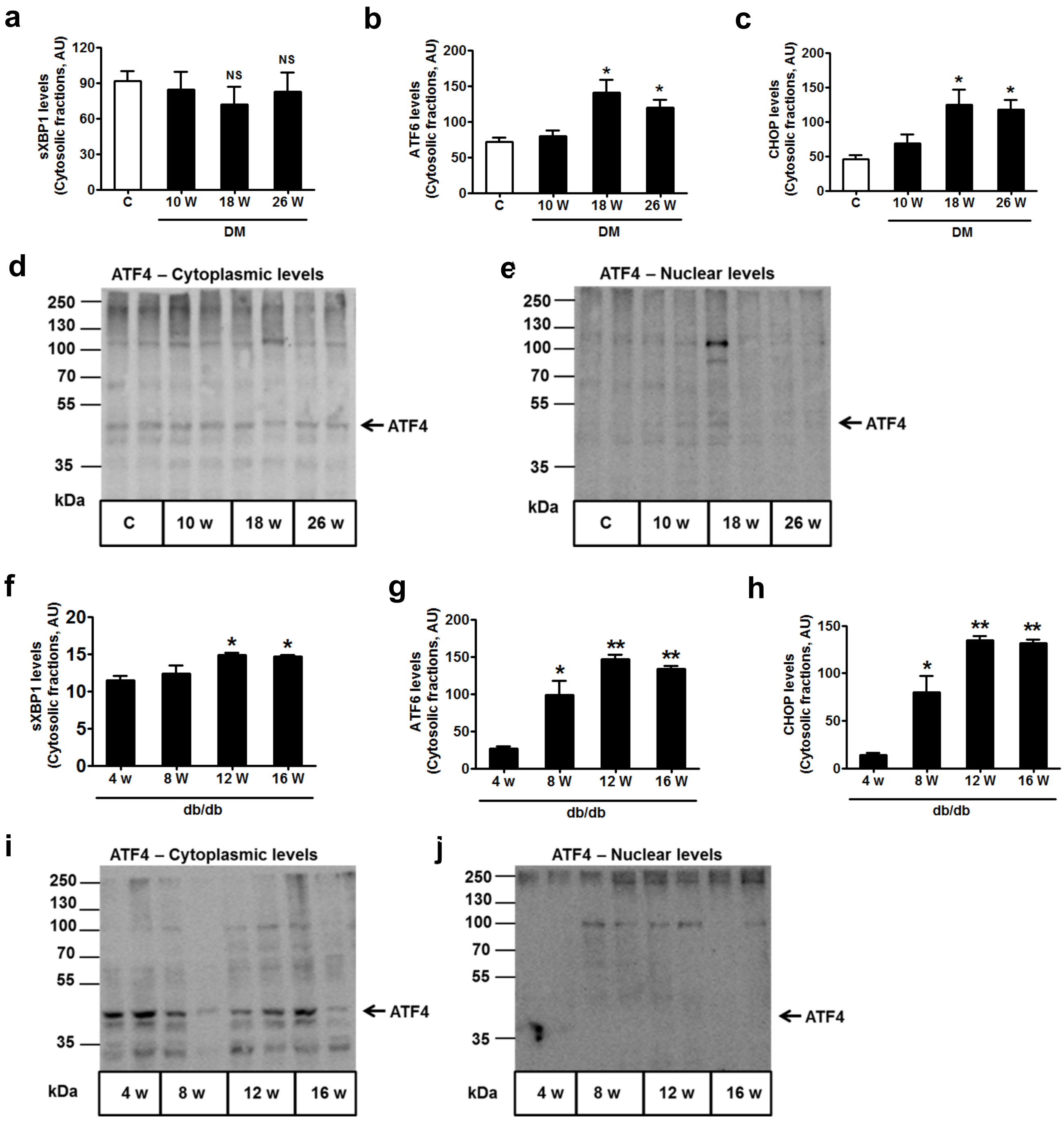

Disparate regulation of renal ER-transcription factors in murine DN

(**a-c, f-h**) Bar graphs showing cytoplasmic levels of ER transcription factors sXBP1 (**a,f**), ATF6 (**b,g**) and CHOP (**c,h**) in renal cortex samples of wild type (C) and STZ-induced diabetic mice (DM) at indicated time points post STZ administration **(a-c)** or in db/db mice at ages indicated (**f-h**).

(**d,e,i,j**) Representative immunoblots showing cytoplasmic levels (**d,i**) and nuclear levels (**e,j**) of ATF4 in renal cortex samples of wild type (C) and STZ-induced diabetic mice (DM) at indicated time points post STZ administration **(d,e)** or in db/db mice at ages indicated (**I,j**). Cytoplasmic levels of ATF4 remain unchanged and nuclear levels undetectable. kDa: kilodalton; w: weeks; Mean ± SEM (a-c; f-h) (n = 6 mice per group).

Supplementary Figure. 2

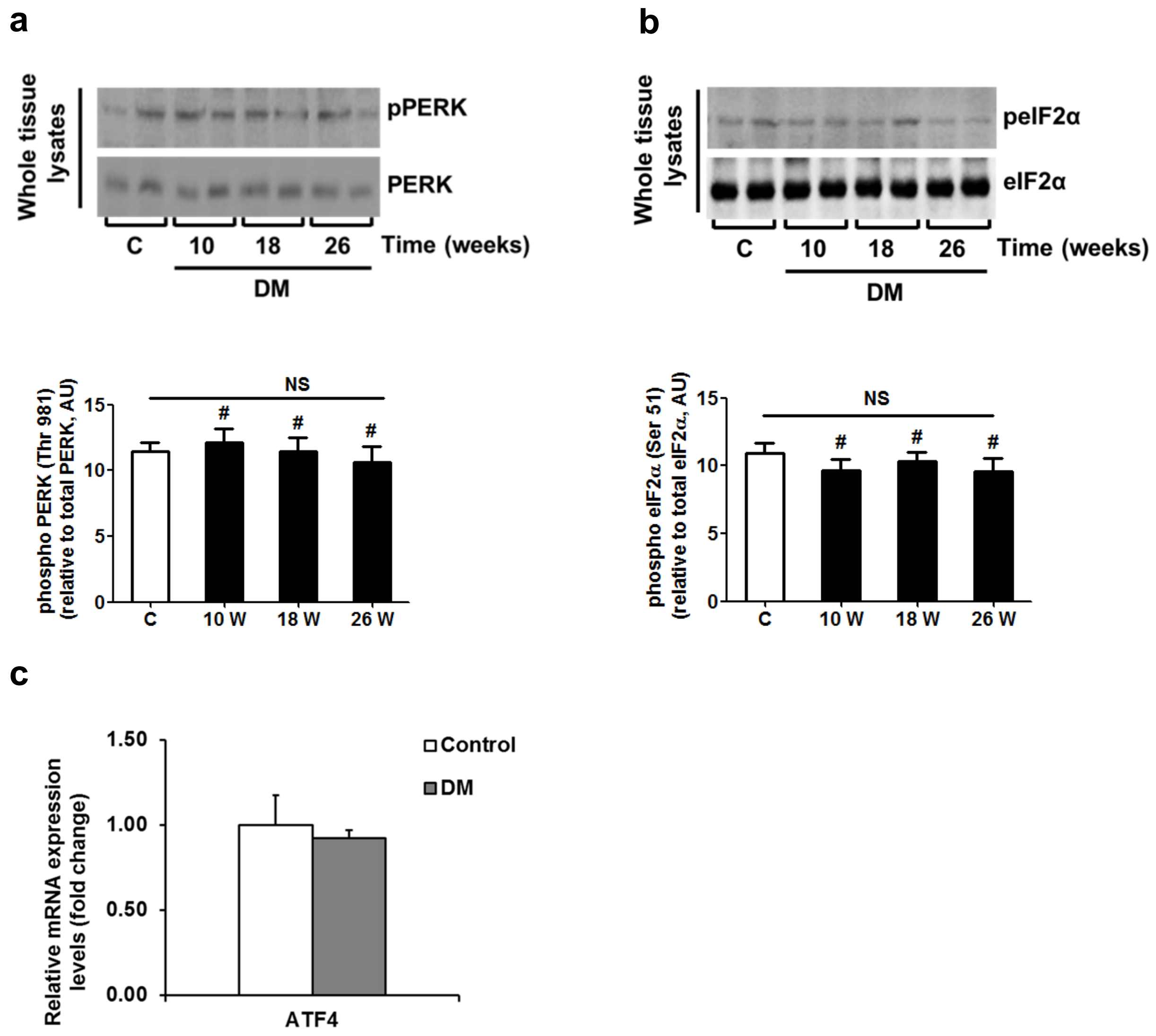

Regulation of PERK-eIF2α activity and ATF4 expression in murine DN

(**a-b**) Representative immunoblots showing phosphorylated and total PERK (a, upper panel) and phosphorylated and total eIF2α (b, upper panel) and in renal cortex samples of wild type (C) and STZ-induced diabetic mice (DM) at indicated time points post STZ administration **(a-b).**

Bar graphs showing phosphorylated levels of PERK(a, lower panel) and phosphorylated levels of eIF2α (b, lower panel) in renal cortex samples of wild type (C) and STZ-induced diabetic mice (DM) at indicated time points post STZ administration.

(c) Bar graph showing Q-RTPCR analysis of ATF4 mRNA levels in wild type (C) and STZ-induced diabetic mice (DM) at 26 weeks post STZ administration.

w: weeks; Mean ± SEM (a-c) of (n = 6 mice per group).

Supplementary Figure. 3

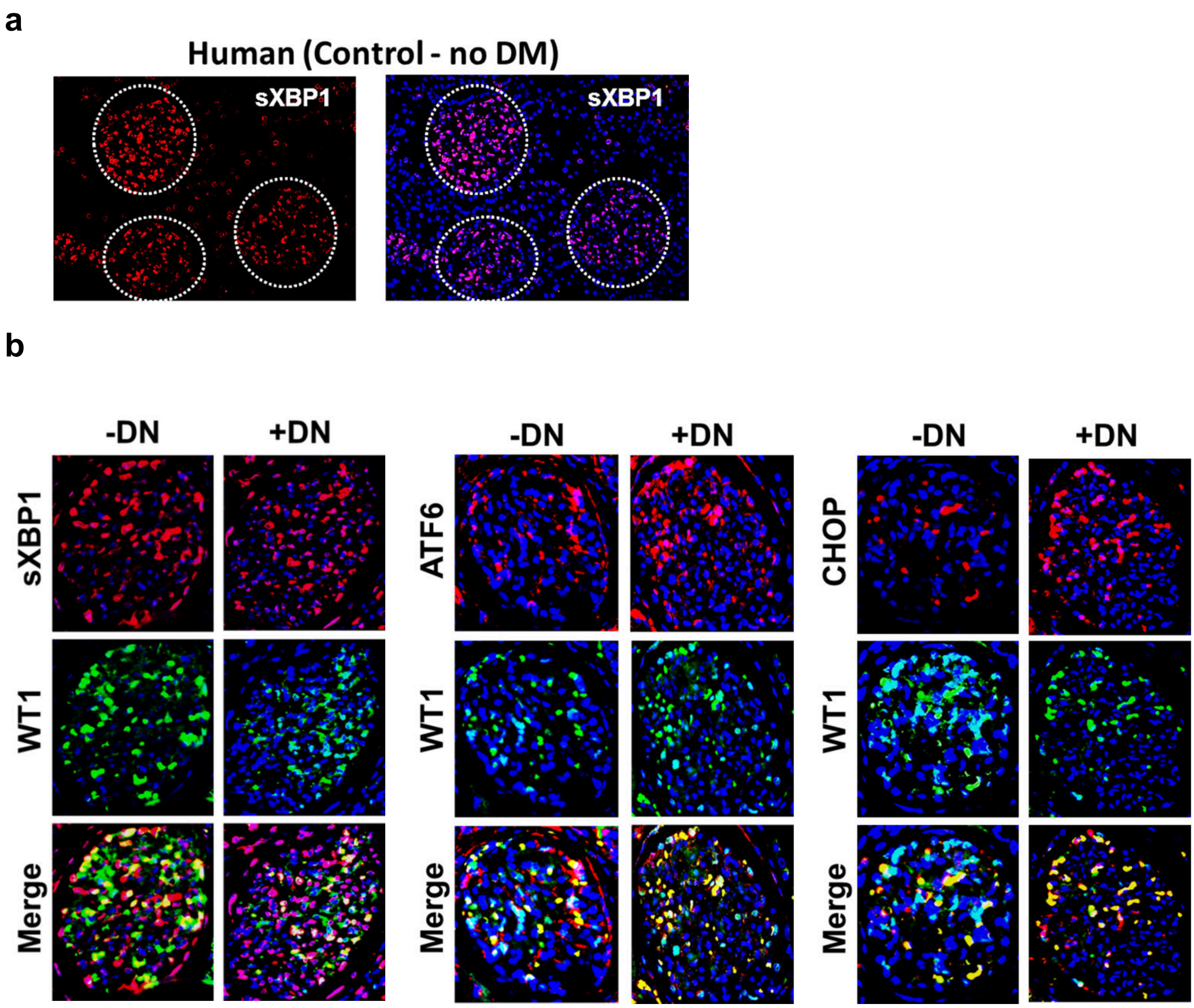

Disparate regulation of renal ER-transcription factors in human DN

(**a**) Representative image at low magnification showing immunofluorescent detection of sXBP1 in a non-diabetic human renal biopsy. sXBP1 (red) is predominately observed in glomeruli (white dotted line) and only sparsely detected in the tubulointerstitial space.

(**b**) Representative immunofluorescent images of human renal biopsies obtained from diabetic patients without (-DN) or with (+DN) diabetic nephropathy, stained for sXBP1, ATF6, CHOP (red) and podocyte marker WT1 (green) with a nuclear counterstain (DAPI, blue). In human diabetic nephropathy, nuclear localization of sXBP1 was reduced primarily in renal glomeruli, while nuclear localization of ATF6 and CHOP were increased when compared to biopsies from healthy subjects.

Supplementary Figure. 4

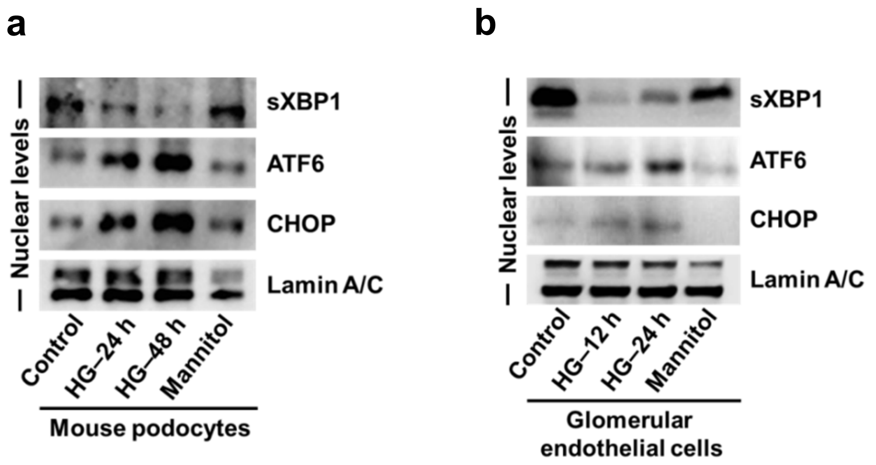

High glucose but not mannitol induce ER-stress in podocytes and glomerular endothelial cells

Representative immunoblots showing nuclear levels of ER transcription factors in immortalized mouse podocytes (**a**) and mouse glomerular endothelial cells (**b**) after treatment with high glucose (HG 25mM) or mannitol (25 mM) at indicated time points. Representative immunoblots of three independent repeat experiments are shown.

Supplementary Figure. 5

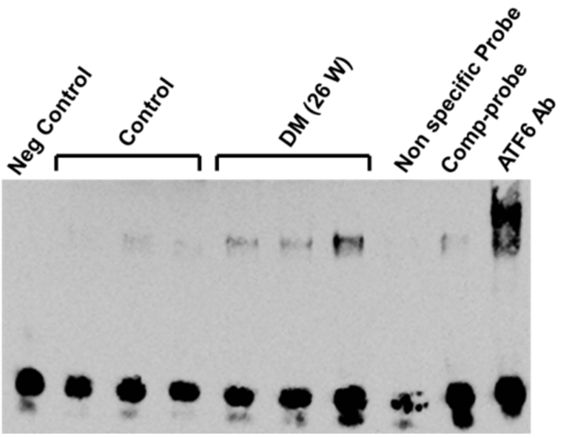

Binding of ATF6 to the CHOP promoter sequence is enhanced in kidneys of diabetic mice
Representative image showing binding of ATF6 to CHOP promoter in nuclear lysates of wild type (Control – non diabetic) and STZ-induced diabetic mice (DM, 26 weeks (W) of persistent hyperglycemia) as analyzed by chemiluminiscent-electrophoretic mobility shift assay. Neg Control: Negative control; Comp probe: competetive unlabeled probe to block binding of labelled probe to the CHOP promoter in a sample obtained from diabetic mice; ATF6 Ab: ATF6 antibody used for supershift.

Supplementary Figure. 6

a

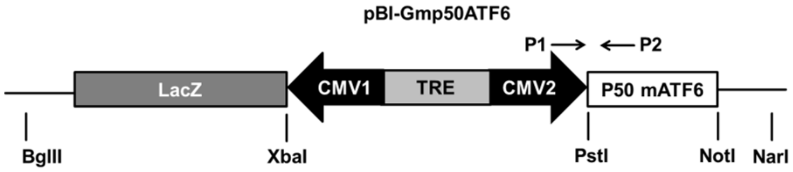

b

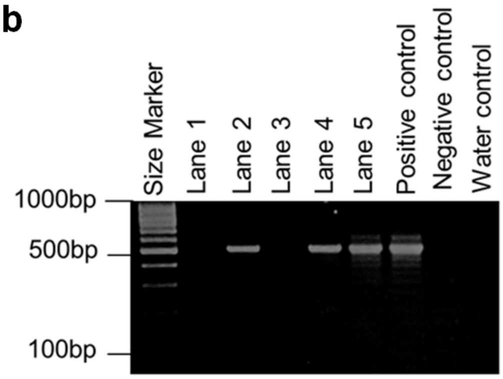

(**a**) Scheme of the expression construct used to generate TETATF6 mice. Mouse P50 ATF6 DNA fragment was amplified by PCR and two restriction sites (PstI and NotI) were introduced. The DNA fragment was digested with PstI and NotI and inserted into corresponding restriction sites of the plasmid pBI-G, which contains a bidirectional TET responsive promoter (clontech).

(**b**) Mice carrying the transgene were identified using the primers P1 (5´-CTCCCACCGTACACGCCTACTCG-3´) and P2 (5´-TCATCTGGTCCATGAGGAAG -3´), yielding a 570 bp fragment. Exemplary image of an agarose gel, lanes 1 and 3 were transgene negative, lanes 2, 4, and 5 were transgene positive.

Supplementary Figure. 7

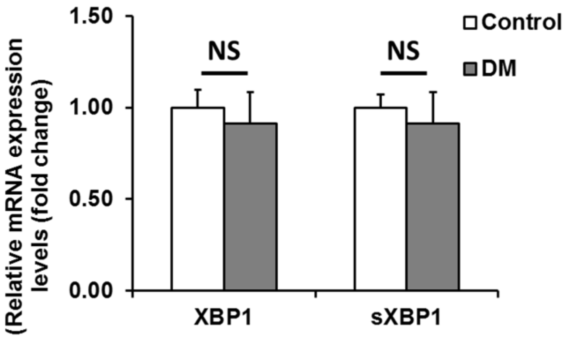

Gene expression of XBP1 and sXBP1 in streptozotocin induced DN
Bar graph showing relative mRNA expression levels of total (XBP1) and spliced (sXBP1) levels in renal cortex samples from untreated (white bars) and STZ-treated (grey bars) mice. Data summarizes Mean ± SEM of 5 independent mice per group. NS: not significant.

Supplementary Figure. 8

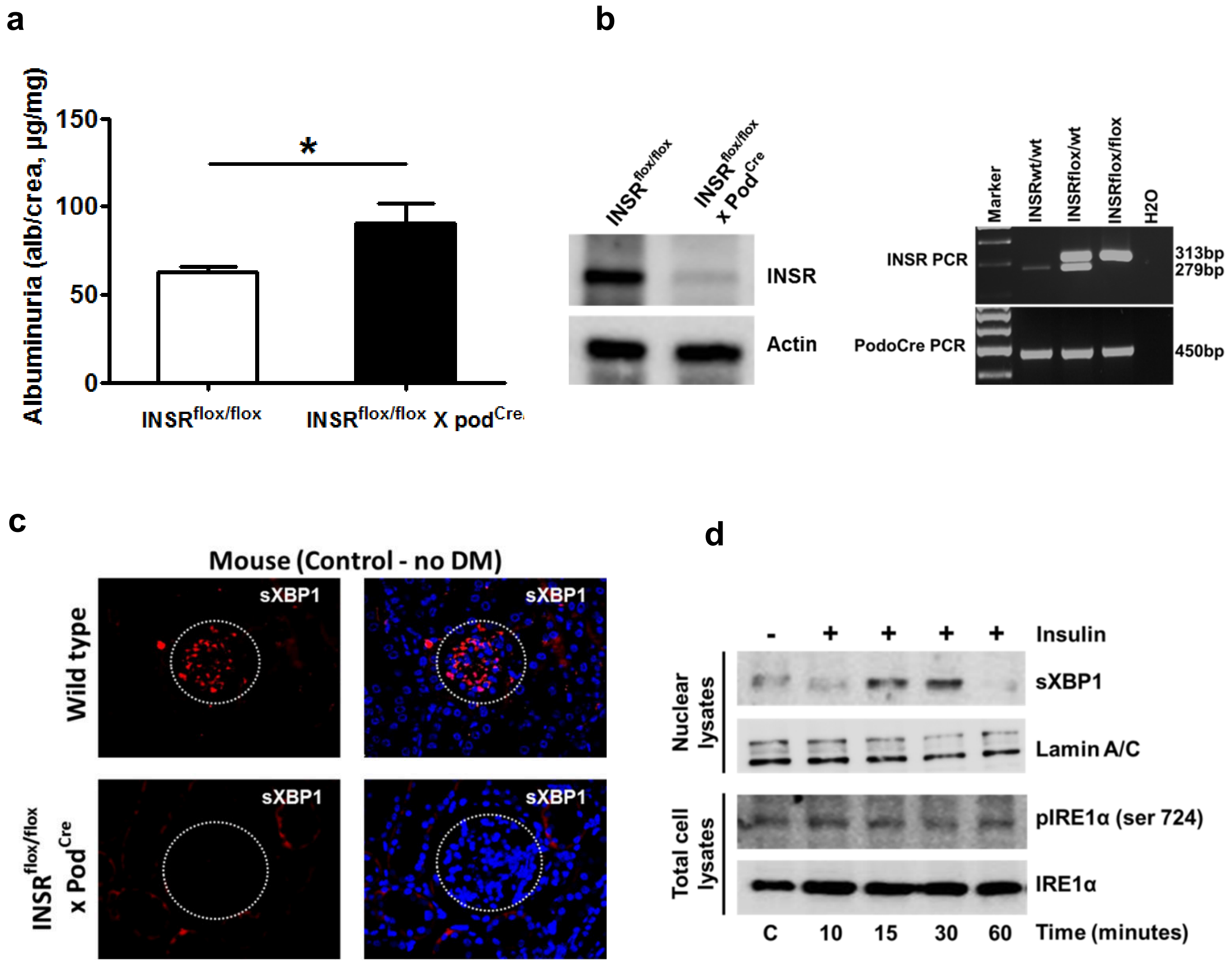

Insulin regulates sXBP1 activity in podocytes

(**a**) Mild but significant spontaneous albuminuria in mice with homozygous podocyte specific INSR deletion. Bar graph reflecting albuminuria in mice with podocyte-specific deletion of insulin receptor ($INSR^{flox/flox}$ x Pod^{Cre}) at 8 weeks of age (N = 8). Mean ± SEM, ★: *P*<0.05 (t-test).

(**b**) Representative image showing PCR analysis of $INSR^{flox/flox}$ x Pod^{Cre} mice (left panel, immunoblot) and tail DNA (right panel) showing nearly complete deletion of insulin receptor (INSR) in podocytes isolated from $INSR^{flox/flox}$ x Pod^{Cre} mice when compared to podocytes isolated from control mice ($INSR^{flox/flox}$).

(**c**) Representative images at low magnification showing renal immunofluorescent detection of sXBP1 in control mice (wild type, top panel) and mice with podocyte specific deletion of insulin receptor in podocytes ($INSR^{flox/flox}$ x Pod^{Cre}, lower panel). sXBP1 (red) is predominately observed in glomeruli (white dotted line) of wild-type mice, but not of $INSR^{flox/flox}$ x Pod^{Cre} mice. sXBP1 is only sparsely detected in the tubulointerstitial space.

(**d**) Insulin promotes nuclear translocation of sXBP1 in mouse podocytes independent of IRE1α phosphorylation. Representative immunoblot showing nuclear levels of sXBP1 and total levels of IRE1α and phosphorylated form of IRE1α (ser-724) after treatment with insulin (100 nM) at indicated time points. Lamin A/C as loading control for nuclear lysates.

Supplementary Figure. 9

Figure. 1e (Nuclear lysates: STZ treated mice)

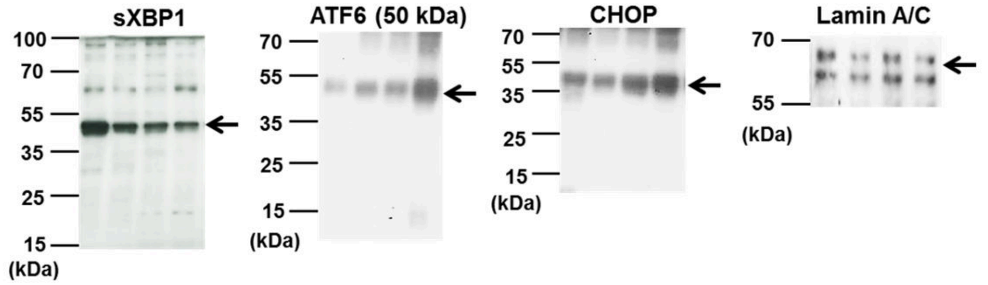

Figure. 1h (Nuclear lysates: db/db mice)

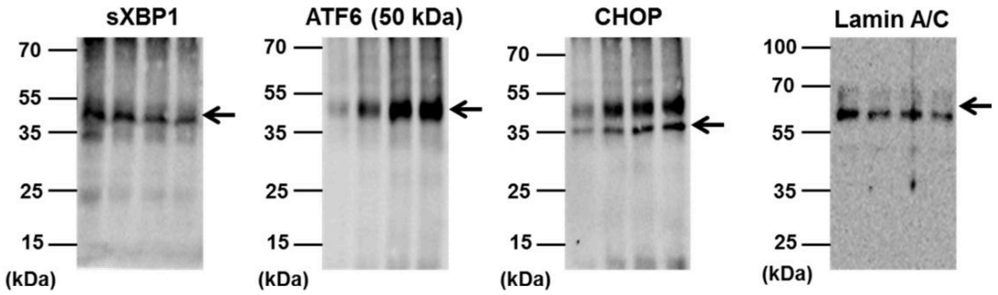

Figure. 3f (Nuclear lysates)

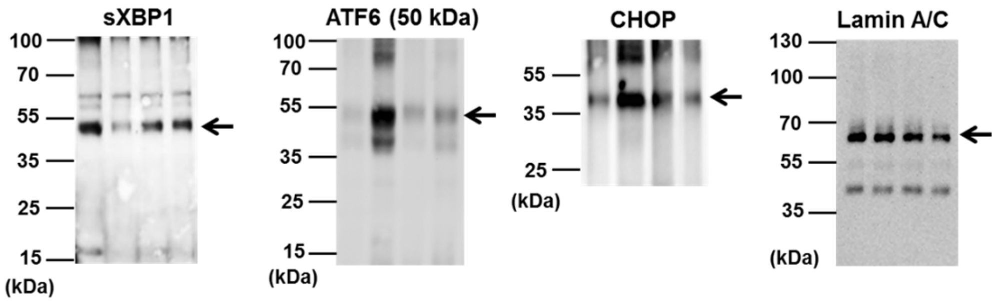

Figure. 4a: podocytes - Cytoplasm

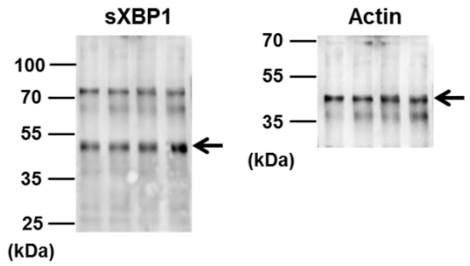

Figure. 4a: GENCs - Cytoplasm

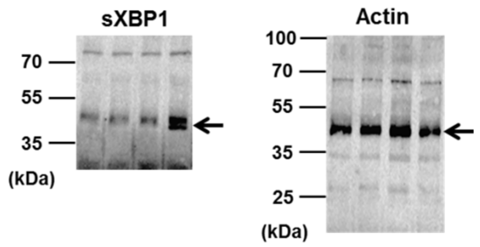

Figure. 4a: podocytes (Nuclear lysates)

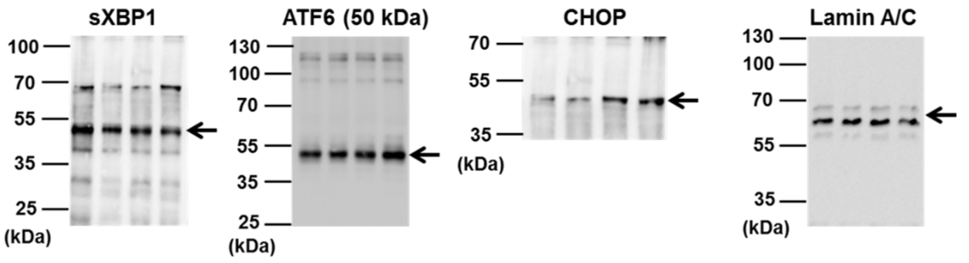

Uncropped scans of the key immunoblot results

Supplementary Figure. 10

Figure. 4a: GENCs (Nuclear lysates)

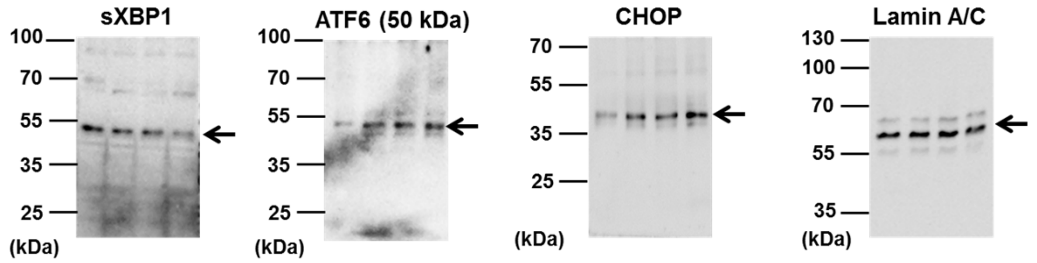

Figure. 4e

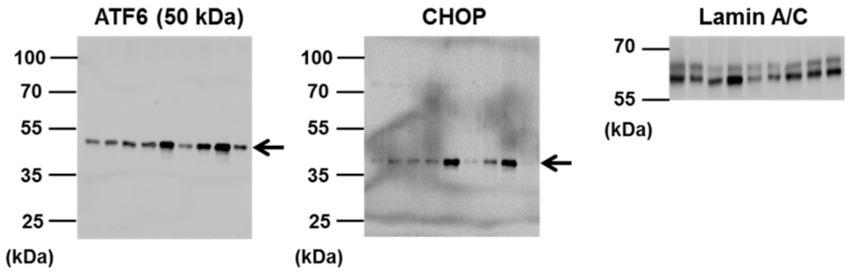

Figure. 5g

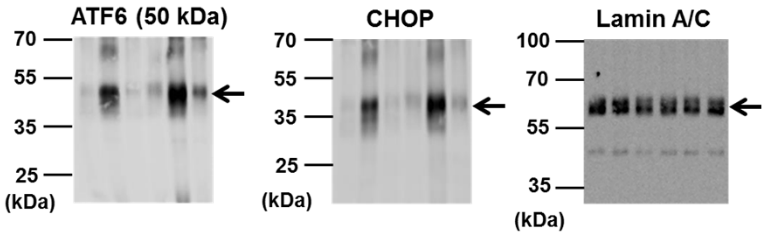

Figure. 6c

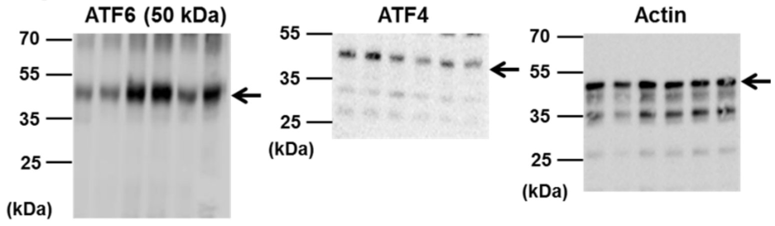

Figure. 6i

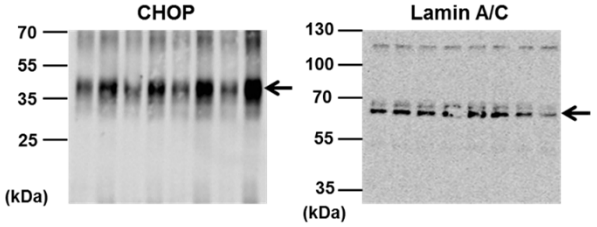

Figure. 8g

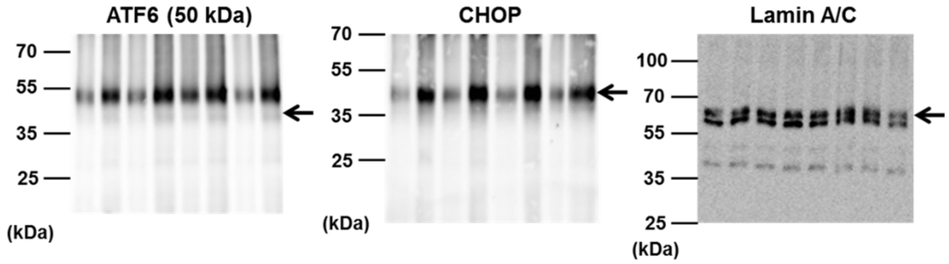

Uncropped scans of the key immunoblot results
